# Crystal structures and comparisons of potassium rare-earth molybdates K*RE*(MoO_4_)_2_ (*RE* = Tb, Dy, Ho, Er, Yb, and Lu)

**DOI:** 10.1107/S205698902001542X

**Published:** 2020-11-27

**Authors:** Saehwa Chong, Samuel Perry, Brian J. Riley, Zayne J. Nelson

**Affiliations:** a Pacific Northwest National Laboratory, Richland, WA 99354, USA; b Department of Civil and Environmental Engineering and Earth Sciences, University, of Notre Dame, Notre Dame, IN 46556, USA

**Keywords:** rare-earth molybdate, lanthanide molybdate, single-crystal XRD

## Abstract

Six potassium rare-earth molybdates K*RE*(MoO_4_)_2_ (*RE* = Tb, Dy, Ho, Er, Yb, and Lu) were synthesized by flux-assisted growth in K_2_Mo_3_O_10_. The crystal structures were determined using single-crystal X-ray diffraction data. The synthesized molybdates crystallize with the ortho­rhom­bic *Pbcn* space group (No. 60). Trendlines for unit-cell parameters were calculated using data from the current study. The unit-cell parameters *a* and *c* increase linearly whereas *b* decreases with larger *RE* cations, based on crystal radii. The unit-cell volumes increase linearly and the densities decrease linearly with larger *RE* cations. The average distances between the *RE* cations and the nearest O atoms increase with larger cations whereas the average distances of Mo—O and K—O do not show specific trends.

## Abstract 2

Six potassium rare-earth molybdates K*RE*(MoO_4_)_2_ (*RE* = Tb, Dy, Ho, Er, Yb, and Lu) were synthesized by flux-assisted growth in K_2_Mo_3_O_10_. The crystal structures were determined using single-crystal X-ray diffraction data. The synthesized molybdates crystallize within the ortho­rhom­bic *Pbcn* space group (No. 60). The unit-cell parameters *a* and *c* increase linearly whereas *b* decreases with larger *RE* cations, based on crystal radii. The unit-cell volumes increase linearly and the densities decrease linearly with larger *RE* cations.

## Chemical context   

Rare-earth (*RE*) molybdates have been studied extensively because of their luminescent, magnetoelectric, and ferroelectric properties (Borchardt & Bierstedt, 1967 ▸; Axe *et al.*, 1971 ▸; Pratap *et al.*, 1987 ▸; Ponomarev *et al.*, 1994 ▸; Shi *et al.*, 1996 ▸; Kut’ko, 2005 ▸; Wang *et al.*, 2007 ▸; Ponomarev & Zhukov, 2012 ▸). The *RE* molybdates of *ARE*(MoO_4_)_2_ (*A* = Li, Na, K, Rb, Cs, Ag) generally crystallize with the tetra­gonal *I*4_1_/*a* space group with the scheelite (CaWO_4_) structure or the ortho­rhom­bic *Pbcn* space group (Wanklyn & Wondre, 1978 ▸; Hanuza & Fomitsev, 1980 ▸; Leask *et al.*, 1981 ▸; Hanuza *et al.*, 1994 ▸; Stedman *et al.*, 1994 ▸; Shi *et al.*, 1996 ▸; Voron’ko *et al.*, 2004 ▸; Kut’ko, 2005 ▸; Wang *et al.*, 2007 ▸; Mat’aš *et al.*, 2010 ▸; Poperezhai *et al.*, 2017 ▸). The *ARE*(MoO_4_)_2_ compounds having the *I*4_1_/*a* space group have luminescent properties with high thermal and hydrolytic stability (Stedman *et al.*, 1994 ▸; Shi *et al.*, 1996 ▸; Voron’ko *et al.*, 2004 ▸; Wang *et al.*, 2007 ▸) whereas the compounds with the *Pbcn* space group are known for the structural phase transition by the Jahn–Teller effect (Kut’ko, 2005 ▸; Mat’aš *et al.*, 2010 ▸; Kamenskyi *et al.*, 2014 ▸; Poperezhai *et al.*, 2017 ▸). Other well-known *RE* molybdates *RE*_2_(MoO_4_)_3_ (*RE* = La, Ce, Pr, Nd, Sm, Eu, Gd, Tb, Dy) crystallize with different space groups including *P*2_1_/*c*, *C*2/*c*, or *P*2*m* depending on the *RE* cations and the synthesis conditions (Brixner *et al.*, 1979 ▸; Jeitschko, 1973 ▸; Ponomarev & Zhukov, 2012 ▸; Pratap *et al.*, 1987 ▸); these phases exhibit magnetoelectric and ferroelectric properties (Borchardt & Bierstedt, 1967 ▸; Axe *et al.*, 1971 ▸; Ponomarev *et al.*, 1994 ▸; Ponomarev & Zhukov, 2012 ▸). The *RE* molybdate compounds are synthesized using flux-assisted or solid-state synthesis methods. Wanklyn & Wondre (1978 ▸) synthesized K*RE*(MoO_4_)_2_ (*RE* = La, Pr, Nd, Sm, Eu, Gd, Tb, Dy, Ho, Er, Lu) compounds by the flux-assisted method using *RE*O_*x*_, MoO_3_, and K_2_SO_4_ at 1000°C for 24 h. They reported that crystals containing *RE* = Tb → Lu crystallized in the *Pbcn* space group whereas *RE* = La and Pr crystallized in the *I*4_1_/*a* space group and others were not defined (Wanklyn & Wondre, 1978 ▸). Shi *et al.* (1996 ▸) synth­esized the Ag*RE*(MoO_4_)_2_ (*RE* = Eu, Gd, Tb) compounds with a tetra­gonal scheelite-type structure by heating the stoichiometric mixtures of *RE*O_*x*_, Ag_*2*_O, and MoO_3_ at 800°C for 50 h. Wang *et al.* (2007 ▸) synthesized the tetra­gonal *A*Eu(MoO_4_)_2_ (*A* = Li, Na, K) compounds by heating a mixture of *RE*O_*x*_, LiCO_3_, NaHCO_3_, K_2_CO_3_, and (NH_4_)_6_Mo_7_O_24_·4H_2_O at 550–750°C for 4 h. The *RE*_2_(MoO_4_)_3_ compounds were synthesized by heating a mixture of *RE*O_*x*_ and MoO_3_ at 900–1000°C (Borchardt & Bierstedt, 1967 ▸; Guzmán-Afonso *et al.*, 2013 ▸).

## Structural commentary   

**Figure 1:**
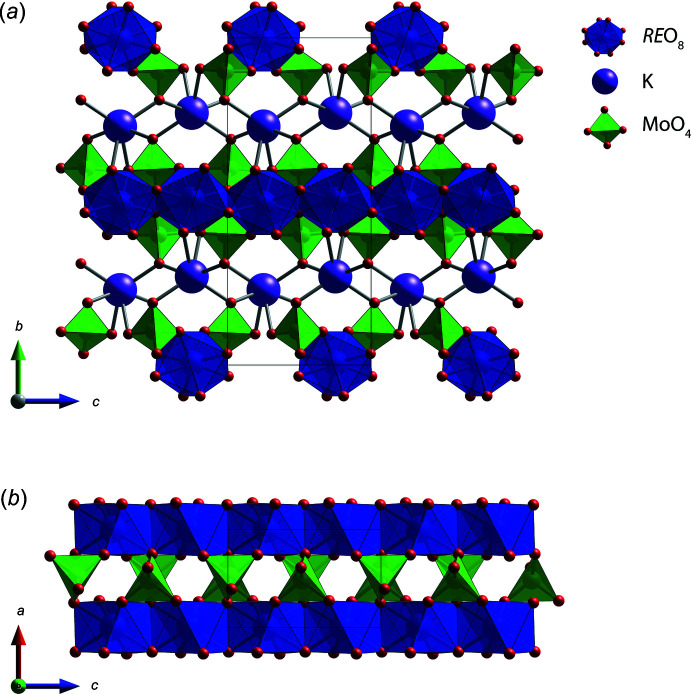
(*a*) Crystal structure of K*RE*(MoO_4_)_2_ and (*b*) [*RE*(MoO_4_)_2_]^−^ layer showing the chains composed of *RE*O_8_ octa­hedra connected by MoO_4_ tetra­hedra along the *c-*axis direction.

**Figure 2:**
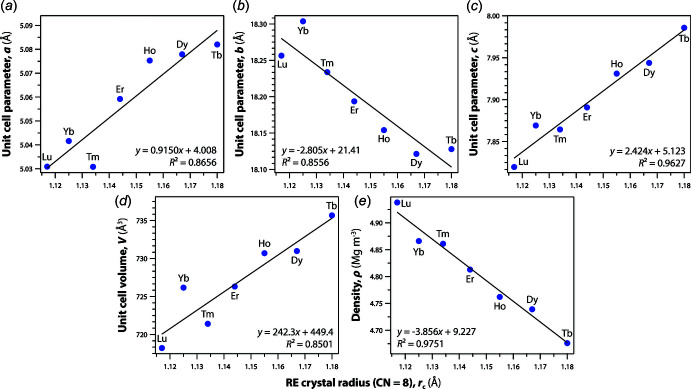
Summary of (*a*) unit-cell parameter *a*, (*b*) unit-cell parameter *b*, (*c*) unit-cell parameter *c*, (*d*) unit-cell volume (*V*), and (*e*) density (*ρ*) as a function of the average crystal radii of the *RE* in the crystal structures (coordination number = 8) from Shannon (1976 ▸).

**Figure 3:**
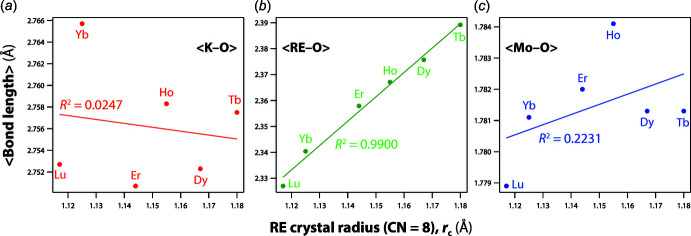
Average distances of (*a*) <K—O>, (*b*) <*RE*—O>, and (*c*) <Mo—O> of K*RE*(MoO_4_)_2_ compounds.

The title K*RE*(MoO_4_)_2_ compounds crystallized in the ortho­rhom­bic *Pbcn* space group and have alternating layers of [*RE*(MoO_4_)_2_]^−^ and K^+^ ions (Fig. 1 ▸ *a*). The [*RE*(MoO_4_)_2_]^−^ layer contains chains formed by edge-sharing *RE*O_8_ octa­hedra connected by MoO_4_ tetra­hedra along the *c*-axis direction (Fig. 1 ▸ *b*). The trendlines of the structural parameters were calculated using data from the current study. The unit-cell parameters of *a* and *c* increase while those of *b* decrease linearly with increasing size of the *RE* cations (Fig. 2 ▸). Although these trends are shown clearly, there are large deviations from the trendlines for some molybdates including Tb and Tm molybdates for unit-cell parameter *a*, and Tb and Yb for unit-cell parameter *b*. The unit-cell volume of the Yb compound also shows a large deviation. Compared to the structural parameters from the previous studies (PDF 00-050-1762 and PDF 00-052-1688) on the Yb compound, the cell length *b* is longer by ∼0.02 Å, and the unit-cell volume is larger by ∼2 Å^3^. The structural parameters in these previous studies are from powder samples whereas the data in this study are from single-crystal studies. These inconsistencies could be due to possible mixed valences of *RE* or non-stoich­iometry of *RE* sites. However, the bond-valence calculations for all the K*RE*(MoO_4_)_2_ compounds show that the bond-valence sums of *RE* cations are close to 3 (Table 1 ▸). The average distances between the *RE* cations and neighboring O atoms increase with larger *RE* cations whereas there are no trends for <Mo—O> or <K—O> (Fig. 3 ▸). Further investigation should be done in the future to understand these deviations from the law. The unit-cell volumes increase linearly whereas the densities of the phases decrease linearly as the radius of the *RE* cations increases (Fig. 2 ▸).

## Synthesis and crystallization   

**Figure 4:**
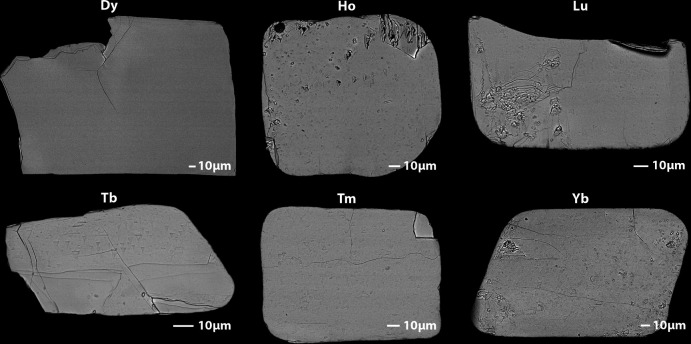
SEM micrographs of K*RE*(MoO_4_)_2_. Artifacts on the surface of several crystals shown resemble residual flux not fully removed during rinsing.

**Figure 5:**
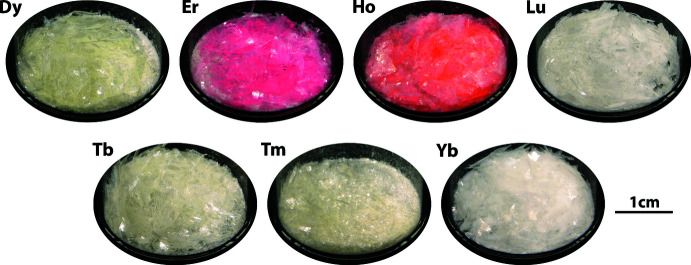
Pictures of recovered crystals of K*RE*(MoO_4_)_2_ showing various sizes and colors.

The single crystals of K*RE*(MoO_4_)_2_ were synthesized using Tb_4_O_7_ (Alfa Aesar, 99.9%), Dy_2_O_3_ (Alfa Aesar, 99.9%), Ho_2_O_3_ (Alfa Aesar, 99.9%), Er_2_O_3_ (Alfa Aesar, 99.9%), Yb_2_O_3_ (Alfa Aesar, 99.9%), Lu_2_O_3_ (Alfa Aesar, 99.9%), K_2_CO_3_ (Alfa Aesar, 99%), and MoO_3_ (Alfa Aesar, 99.5%). All the chemicals were used as received. First, K_2_Mo_3_O_10_ was synthesized using K_2_CO_3_ and MoO_3_ by heating at 520°C for 8 h as described in a previous study (Chong *et al.*, 2020 ▸). The stoichiometric mixture of *RE*O_*x*_ and K_2_Mo_3_O_10_ was put into a Pt/10%Rh crucible with a lid and placed in a Thermolyne box furnace. The furnace was heated to 1150°C at 5°C min^−1^, dwelled for 10 h, cooled to 400°C at 5°C h^−1^ in air, and then shut off. The single crystals were recovered from the solidified flux after washing in an ultrasonic bath with deionized water and using vacuum filtration. In addition to the listed six crystals, KTm(MoO_4_)_2_ was synthesized similarly, but it was not reported in this study due to the unresolved residual electron densities during structural refinement. All the K*RE*(MoO_4_)_2_ crystals were plates (Fig. 4 ▸) with different colors (Fig. 5 ▸).

## Refinement   

Crystal data, data collection and structure refinement details are summarized in Table 2 ▸. A hemisphere of data was collected on two crystals (*RE* = Ho, Yb) using a Bruker APEXII Qu­azar diffractometer equipped with a microsource tube emitting monochromated Mo K*α* X-ray radiation and collected on a CCD detector. Data were collected for five crystals (*RE* = Dy, Er, Lu, Tb, Tm) with a Rigaku XtaLab Synergy diffractometer using a single microfocus Mo K*α* X-ray radiation source in a sealed tube, equipped with a Hybrid Pixel (HyPix) Array detector and using an Oxford liquid-nitro­gen Cryostream. For the Bruker datasets, *APEX3* software (Bruker, 2014 ▸) was used for determining the unit cell and integrating the collected reflection data. Absorption corrections were applied with the *SADABS* software package (Krause *et al.*, 2015 ▸). For the Rigaku datasets, the *CrysAlis Pro* software package was used for unit-cell determination and data integration (Rigaku OD, 2019 ▸). The numerical absorption correction was applied utilizing SCALE3 ABSPACK (Clark & Reid, 1995 ▸). All structures were solved by the intrinsic phasing method using *SHELXT* and refined with *SHELXL* (Sheldrick, 2015*a* ▸,*b* ▸) within the *OLEX2* software package (Dolomanov *et al.*, 2009 ▸).

## Supplementary Material

Crystal structure: contains datablock(s) global, Tb_Molybdate, Dy_Molybdate, Ho_Molybdate, Er_Molybdate, Yb_Molybdate, Lu_Molybdate. DOI: 10.1107/S205698902001542X/ru2071sup1.cif

Structure factors: contains datablock(s) Tb_Molybdate. DOI: 10.1107/S205698902001542X/ru2071Tb_Molybdatesup2.hkl

Structure factors: contains datablock(s) Dy_Molybdate. DOI: 10.1107/S205698902001542X/ru2071Dy_Molybdatesup3.hkl

Structure factors: contains datablock(s) Ho_Molybdate. DOI: 10.1107/S205698902001542X/ru2071Ho_Molybdatesup4.hkl

Structure factors: contains datablock(s) Er_Molybdate. DOI: 10.1107/S205698902001542X/ru2071Er_Molybdatesup5.hkl

Structure factors: contains datablock(s) Yb_Molybdate. DOI: 10.1107/S205698902001542X/ru2071Yb_Molybdatesup6.hkl

Structure factors: contains datablock(s) Lu_Molybdate. DOI: 10.1107/S205698902001542X/ru2071Lu_Molybdatesup7.hkl

Bond-valence tables. DOI: 10.1107/S205698902001542X/ru2071sup8.pdf

CCDC references: 2045648, 2045647, 2045646, 2045645, 2045644, 2045643

Additional supporting information:  crystallographic information; 3D view; checkCIF report

## Figures and Tables

**Table 1:** Bond-valence (v.u.) calculations for the title K*RE*(MoO_4_)_2_ compounds Detailed tables are included in the supporting information.

	KTb(MoO_4_)_2_	KDy(MoO_4_)_2_	KHo(MoO_4_)_2_	KEr(MoO_4_)_2_	KYb(MoO_4_)_2_	KLu(MoO_4_)_2_
*RE*	3.11	2.97	3.25	3.00	2.97	3.13
Mo	5.66	5.66	5.62	5.65	5.66	5.70
K	1.20	1.21	1.19	1.20	1.10	1.13
O_1_	1.99	1.97	2.00	1.98	1.98	2.00
O_2_	1.82	1.80	1.82	1.80	1.79	1.82
O_3_	1.90	1.92	1.97	1.92	1.91	1.91
O_4_	2.06	2.07	2.07	2.05	2.03	2.09

**Table:** 

	KTb(MoO_4_)_2_	KDy(MoO_4_)_2_	KHo(MoO_4_)_2_
Crystal data			
*M*_r_	517.90	521.48	523.91
Crystal system, space group	Orthorhombic, *P* *b* *c* *n*	Orthorhombic, *P* *b* *c* *n*	Orthorhombic, *P* *b* *c* *n*
Temperature (K)	100	100	273
*a*, *b*, *c* (Å)	5.0826 (1), 18.1273 (7), 7.9875 (2)	5.0776 (2), 18.1214 (7), 7.9428 (3)	5.0770 (15), 18.161 (5), 7.934 (2)
*V* (Å^3^)	735.92 (4)	730.84 (5)	731.5 (4)
*Z*	4	4	4
Radiation type	Mo *K*α	Mo *K*α	Mo *K*α
μ (mm^−1^)	13.43	14.07	14.66
Crystal size (mm)	0.36 × 0.20 × 0.04	0.11 × 0.11 × 0.03	0.06 × 0.06 × 0.04

Data collection			
Diffractometer	Rigaku XtaLAB Synergy-S, HyPix	Rigaku XtaLAB Synergy-S, HyPix	Bruker APEXII CCD
Absorption correction	Gaussian (*CrysAlis PRO*; Rigaku OD, 2019 ▸)	Gaussian (*CrysAlis PRO*; Rigaku OD, 2019 ▸)	Multi-scan (*SADABS*; Krause et al., 2015 ▸)
*T*_min_, *T*_max_	0.035, 0.703	0.247, 0.695	0.248, 0.343
No. of measured, independent and observed [*I* > 2σ(*I*)] reflections	13792, 1350, 1238	17317, 1388, 1196	7665, 936, 818
*R*_int_	0.069	0.075	0.032

Refinement			
*R*[*F* ^2^ > 2σ(*F* ^2^)], *wR*(*F* ^2^), *S*	0.033, 0.102, 1.08	0.033, 0.088, 1.10	0.027, 0.063, 1.25
No. of reflections	1350	1388	936
No. of parameters	56	56	56
Δρ_max_, Δρ_min_ (e Å^−3^)	2.63, −2.78	2.38, −2.65	1.51, −1.37

**Table:** 

	KEr(MoO_4_)_2_	KYb(MoO_4_)_2_	KLu(MoO_4_)_2_
Crystal data			
*M*_r_	526.24	532.02	533.95
Crystal system, space group	Orthorhombic, *P* *b* *c* *n*	Orthorhombic, *P* *b* *c* *n*	Orthorhombic, *P* *b* *c* *n*
Temperature (K)	100	273	100
*a*, *b*, *c* (Å)	5.0602 (2), 18.1965 (8), 7.8920 (3)	5.0417 (5), 18.3039 (19), 7.8693 (8)	5.0292 (2), 18.2519 (10), 7.8174 (4)
*V* (Å^3^)	726.68 (5)	726.20 (13)	717.58 (6)
*Z*	4	4	4
Radiation type	Mo *K*α	Mo *K*α	Mo *K*α
μ (mm^−1^)	15.42	16.75	17.68
Crystal size (mm)	0.28 × 0.15 × 0.02	0.06 × 0.06 × 0.04	0.44 × 0.15 × 0.04

Data collection			
Diffractometer	Rigaku XtaLAB Synergy-S, HyPix	Bruker APEXII CCD	Rigaku XtaLAB Synergy-S, HyPix
Absorption correction	Gaussian (*CrysAlis PRO*; Rigaku OD, 2019 ▸)	Multi-scan (*SADABS*; Krause et al., 2015 ▸)	Multi-scan (*CrysAlis PRO*; Rigaku OD, 2019 ▸)
*T*_min_, *T*_max_	0.091, 0.911	0.198, 0.301	0.240, 1.000
No. of measured, independent and observed [*I* > 2σ(*I*)] reflections	21246, 1386, 1255	8164, 978, 845	19857, 1105, 1004
*R*_int_	0.072	0.038	0.087

Refinement			
*R*[*F* ^2^ > 2σ(*F* ^2^)], *wR*(*F* ^2^), *S*	0.027, 0.073, 1.06	0.022, 0.063, 1.11	0.027, 0.077, 1.17
No. of reflections	1386	978	1105
No. of parameters	56	56	57
Δρ_max_, Δρ_min_ (e Å^−3^)	2.04, −1.62	1.55, −1.14	2.05, −3.03

Computer programs: *CrysAlis PRO* (Rigaku OD, 2019 ▸), *APEX3* (Bruker, 2014 ▸), *SHELXT* (Sheldrick, 2015*a* ▸), *SHELXL* (Sheldrick, 2015*b* ▸), *OLEX2* (Dolomanov *et al.*, 2009 ▸), and *publCIF* (Westrip, 2010 ▸).

## supplementary crystallographic information

### Potassium terbium bis(molybdate) (Tb_Molybdate) Crystal data

**Table:**

KTb(MoO_4_)_2_	*F*(000) = 928
*M**_r_* = 517.90	*D*_x_ = 4.674 Mg m^−^^3^
Orthorhombic, *P**b**c**n*	Mo *K*α radiation, λ = 0.71073 Å
Hall symbol: -P 2n 2ab	Cell parameters from 6923 reflections
*a* = 5.0826 (1) Å	θ = 2.3–33.5°
*b* = 18.1273 (7) Å	µ = 13.43 mm^−^^1^
*c* = 7.9875 (2) Å	*T* = 100 K
*V* = 735.92 (4) Å^3^	Plate, white
*Z* = 4	0.36 × 0.20 × 0.04 mm

### Potassium terbium bis(molybdate) (Tb_Molybdate) Data collection

**Table:**

Rigaku XtaLAB Synergy-S, HyPix diffractometer	1350 independent reflections
Radiation source: micro-focus sealed X-ray tube	1238 reflections with *I* > 2σ(*I*)
Mirror monochromator	*R*_int_ = 0.069
Detector resolution: 10.0000 pixels mm^-1^	θ_max_ = 33.7°, θ_min_ = 2.3°
ω scans	*h* = −7→6
Absorption correction: gaussian (CrysAlisPro; Rigaku OD, 2019)	*k* = −27→27
*T*_min_ = 0.035, *T*_max_ = 0.703	*l* = −12→12
13792 measured reflections	

### Potassium terbium bis(molybdate) (Tb_Molybdate) Refinement

**Table:**

Refinement on *F*^2^	0 restraints
Least-squares matrix: full	0 constraints
*R*[*F*^2^ > 2σ(*F*^2^)] = 0.033	Primary atom site location: dual
*wR*(*F*^2^) = 0.102	*w* = 1/[σ^2^(*F*_o_^2^) + (0.0705*P*)^2^ + 1.3389*P*] where *P* = (*F*_o_^2^ + 2*F*_c_^2^)/3
*S* = 1.08	(Δ/σ)_max_ = 0.002
1350 reflections	Δρ_max_ = 2.63 e Å^−^^3^
56 parameters	Δρ_min_ = −2.78 e Å^−^^3^

### Potassium terbium bis(molybdate) (Tb_Molybdate) Special details

**Table:**

Geometry. All esds (except the esd in the dihedral angle between two l.s. planes) are estimated using the full covariance matrix. The cell esds are taken into account individually in the estimation of esds in distances, angles and torsion angles; correlations between esds in cell parameters are only used when they are defined by crystal symmetry. An approximate (isotropic) treatment of cell esds is used for estimating esds involving l.s. planes.
Refinement. Reflections were merged by SHELXL according to the crystal class for the calculation of statistics and refinement. _reflns_Friedel_fraction is defined as the number of unique Friedel pairs measured divided by the number that would be possible theoretically, ignoring centric projections and systematic absences.

### Potassium terbium bis(molybdate) (Tb_Molybdate) Fractional atomic coordinates and isotropic or equivalent isotropic displacement parameters (Å^2^)

**Table:**

	*x*	*y*	*z*	*U*_iso_*/*U*_eq_	
Tb1	0.000000	0.49384 (2)	0.250000	0.00653 (12)	
Mo1	0.48012 (7)	0.60289 (2)	0.48337 (5)	0.00701 (12)	
K1	0.500000	0.77012 (8)	0.750000	0.0114 (2)	
O2	0.7489 (5)	0.53496 (13)	0.5042 (2)	0.0089 (4)	
O4	0.6111 (5)	0.69028 (13)	0.4759 (3)	0.0105 (5)	
O1	0.2592 (5)	0.59503 (12)	0.3093 (3)	0.0099 (4)	
O3	0.2716 (5)	0.60251 (12)	0.6603 (3)	0.0102 (4)	

### Potassium terbium bis(molybdate) (Tb_Molybdate) Atomic displacement parameters (Å^2^)

**Table:**

	*U*^11^	*U*^22^	*U*^33^	*U*^12^	*U*^13^	*U*^23^
Tb1	0.00409 (18)	0.01076 (18)	0.00476 (17)	0.000	−0.00033 (5)	0.000
Mo1	0.00425 (19)	0.01069 (19)	0.00610 (19)	0.00024 (8)	−0.00066 (7)	−0.00020 (10)
K1	0.0118 (5)	0.0136 (5)	0.0089 (5)	0.000	−0.0008 (3)	0.000
O2	0.0056 (12)	0.0138 (9)	0.0073 (9)	0.0017 (9)	−0.0008 (6)	0.0002 (7)
O4	0.0096 (12)	0.0130 (10)	0.0089 (10)	−0.0036 (9)	0.0011 (7)	−0.0007 (8)
O1	0.0069 (10)	0.0143 (10)	0.0084 (10)	−0.0001 (8)	−0.0021 (8)	0.0002 (8)
O3	0.0089 (10)	0.0144 (10)	0.0073 (9)	−0.0003 (8)	0.0003 (8)	−0.0008 (7)

### Potassium terbium bis(molybdate) (Tb_Molybdate) Geometric parameters (Å, º)

**Table:**

Tb1—Tb1^i^	4.0000 (1)	Mo1—O3	1.767 (2)
Tb1—Tb1^ii^	4.0000 (1)	K1—Mo1^xi^	3.8378 (9)
Tb1—K1^iii^	4.2788 (14)	K1—Mo1^xii^	3.7062 (11)
Tb1—O2^iv^	2.399 (2)	K1—Mo1^xiii^	3.8378 (9)
Tb1—O2^v^	2.399 (2)	K1—O4	2.684 (2)
Tb1—O2^vi^	2.511 (2)	K1—O4^xiv^	2.771 (2)
Tb1—O2^vii^	2.511 (2)	K1—O4^xii^	2.684 (2)
Tb1—O1^viii^	2.308 (2)	K1—O4^xv^	2.771 (2)
Tb1—O1	2.308 (2)	K1—O1^xiii^	2.817 (3)
Tb1—O3^ix^	2.339 (2)	K1—O1^xi^	2.817 (3)
Tb1—O3^ii^	2.339 (2)	K1—O3	3.330 (3)
Mo1—K1	3.7062 (11)	K1—O3^xii^	3.330 (3)
Mo1—K1^x^	3.9694 (9)	O2—Tb1^iv^	2.399 (2)
Mo1—K1^iii^	3.8378 (9)	O2—Tb1^xvi^	2.511 (2)
Mo1—O2	1.847 (2)	O4—K1^x^	2.771 (2)
Mo1—O4	1.719 (2)	O1—K1^iii^	2.817 (3)
Mo1—O1	1.793 (2)	O3—Tb1^ii^	2.339 (2)
			
Tb1^ii^—Tb1—Tb1^i^	173.598 (18)	Mo1^xiii^—K1—Mo1^xi^	106.29 (4)
Tb1^ii^—Tb1—K1^iii^	86.799 (9)	Mo1—K1—Mo1^xiii^	103.230 (11)
Tb1^i^—Tb1—K1^iii^	86.799 (9)	Mo1^xii^—K1—Mo1^xiii^	138.798 (13)
O2^iv^—Tb1—Tb1^i^	146.02 (5)	Mo1—K1—Mo1^xii^	70.24 (3)
O2^v^—Tb1—Tb1^i^	36.40 (5)	Mo1^xii^—K1—Mo1^xi^	103.230 (11)
O2^vi^—Tb1—Tb1^i^	34.54 (5)	O4^xii^—K1—Mo1	91.25 (6)
O2^vii^—Tb1—Tb1^i^	142.24 (5)	O4—K1—Mo1^xii^	91.25 (6)
O2^iv^—Tb1—Tb1^ii^	36.40 (5)	O4^xiv^—K1—Mo1^xi^	72.98 (5)
O2^v^—Tb1—Tb1^ii^	146.02 (5)	O4^xiv^—K1—Mo1^xii^	127.27 (6)
O2^vi^—Tb1—Tb1^ii^	142.24 (5)	O4^xii^—K1—Mo1^xi^	78.09 (5)
O2^vii^—Tb1—Tb1^ii^	34.54 (5)	O4—K1—Mo1	25.32 (5)
O2^vi^—Tb1—K1^iii^	72.73 (5)	O4^xiv^—K1—Mo1	79.53 (5)
O2^vii^—Tb1—K1^iii^	72.73 (5)	O4—K1—Mo1^xiii^	78.09 (5)
O2^v^—Tb1—K1^iii^	102.57 (6)	O4—K1—Mo1^xi^	148.56 (6)
O2^iv^—Tb1—K1^iii^	102.57 (6)	O4^xii^—K1—Mo1^xiii^	148.57 (6)
O2^vii^—Tb1—O2^vi^	145.47 (11)	O4^xv^—K1—Mo1^xi^	88.96 (6)
O2^iv^—Tb1—O2^vi^	117.12 (10)	O4^xv^—K1—Mo1^xiii^	72.98 (5)
O2^iv^—Tb1—O2^vii^	70.93 (9)	O4^xii^—K1—Mo1^xii^	25.32 (5)
O2^v^—Tb1—O2^vi^	70.93 (9)	O4^xiv^—K1—Mo1^xiii^	88.96 (6)
O2^v^—Tb1—O2^vii^	117.12 (10)	O4^xv^—K1—Mo1	127.27 (6)
O2^v^—Tb1—O2^iv^	154.87 (11)	O4^xv^—K1—Mo1^xii^	79.53 (5)
O1—Tb1—Tb1^i^	99.23 (6)	O4^xii^—K1—O4^xv^	76.03 (6)
O1^viii^—Tb1—Tb1^i^	75.57 (6)	O4—K1—O4^xv^	121.38 (3)
O1—Tb1—Tb1^ii^	75.57 (6)	O4^xv^—K1—O4^xiv^	149.97 (11)
O1^viii^—Tb1—Tb1^ii^	99.23 (6)	O4—K1—O4^xiv^	76.03 (6)
O1^viii^—Tb1—K1^iii^	37.35 (6)	O4^xii^—K1—O4^xiv^	121.38 (3)
O1—Tb1—K1^iii^	37.35 (6)	O4—K1—O4^xii^	114.75 (11)
O1^viii^—Tb1—O2^iv^	130.14 (8)	O4^xii^—K1—O1^xi^	103.44 (7)
O1—Tb1—O2^vi^	68.88 (7)	O4—K1—O1^xi^	134.69 (7)
O1^viii^—Tb1—O2^vii^	68.88 (7)	O4—K1—O1^xiii^	103.44 (7)
O1^viii^—Tb1—O2^vi^	83.59 (8)	O4^xiv^—K1—O1^xiii^	89.97 (8)
O1—Tb1—O2^iv^	72.62 (8)	O4^xiv^—K1—O1^xi^	63.32 (7)
O1—Tb1—O2^vii^	83.59 (8)	O4^xii^—K1—O1^xiii^	134.69 (7)
O1^viii^—Tb1—O2^v^	72.62 (8)	O4^xv^—K1—O1^xiii^	63.32 (7)
O1—Tb1—O2^v^	130.14 (8)	O4^xv^—K1—O1^xi^	89.97 (8)
O1—Tb1—O1^viii^	74.71 (11)	O4^xv^—K1—O3	128.69 (7)
O1^viii^—Tb1—O3^ix^	150.21 (8)	O4^xv^—K1—O3^xii^	81.24 (7)
O1^viii^—Tb1—O3^ii^	108.63 (9)	O4^xii^—K1—O3	67.06 (7)
O1—Tb1—O3^ix^	108.63 (9)	O4^xiv^—K1—O3	81.24 (7)
O1—Tb1—O3^ii^	150.21 (8)	O4^xiv^—K1—O3^xii^	128.69 (7)
O3^ix^—Tb1—Tb1^i^	74.69 (5)	O4—K1—O3^xii^	67.06 (7)
O3^ix^—Tb1—Tb1^ii^	110.34 (5)	O4—K1—O3	53.56 (7)
O3^ii^—Tb1—Tb1^ii^	74.69 (5)	O4^xii^—K1—O3^xii^	53.56 (7)
O3^ii^—Tb1—Tb1^i^	110.34 (5)	O1^xiii^—K1—Mo1	128.75 (5)
O3^ii^—Tb1—K1^iii^	138.32 (6)	O1^xiii^—K1—Mo1^xi^	81.87 (6)
O3^ix^—Tb1—K1^iii^	138.32 (6)	O1^xi^—K1—Mo1^xii^	128.75 (5)
O3^ix^—Tb1—O2^vii^	140.29 (7)	O1^xi^—K1—Mo1	142.52 (5)
O3^ix^—Tb1—O2^iv^	76.95 (8)	O1^xiii^—K1—Mo1^xii^	142.52 (5)
O3^ii^—Tb1—O2^vii^	70.98 (8)	O1^xiii^—K1—Mo1^xiii^	25.90 (5)
O3^ii^—Tb1—O2^v^	76.95 (8)	O1^xi^—K1—Mo1^xiii^	81.87 (6)
O3^ix^—Tb1—O2^vi^	70.98 (8)	O1^xi^—K1—Mo1^xi^	25.90 (5)
O3^ii^—Tb1—O2^vi^	140.29 (7)	O1^xiii^—K1—O1^xi^	59.61 (10)
O3^ii^—Tb1—O2^iv^	84.31 (8)	O1^xiii^—K1—O3^xii^	131.66 (6)
O3^ix^—Tb1—O2^v^	84.31 (8)	O1^xi^—K1—O3	131.66 (6)
O3^ii^—Tb1—O3^ix^	83.37 (11)	O1^xi^—K1—O3^xii^	156.71 (6)
K1—Mo1—K1^x^	77.135 (10)	O1^xiii^—K1—O3	156.71 (6)
K1^iii^—Mo1—K1^x^	81.22 (2)	O3—K1—Mo1^xiii^	131.63 (4)
K1—Mo1—K1^iii^	78.802 (10)	O3—K1—Mo1	28.44 (4)
O2—Mo1—K1^iii^	156.08 (6)	O3^xii^—K1—Mo1^xi^	131.63 (4)
O2—Mo1—K1^x^	86.36 (7)	O3^xii^—K1—Mo1^xiii^	115.47 (4)
O2—Mo1—K1	118.26 (7)	O3^xii^—K1—Mo1	52.19 (4)
O4—Mo1—K1^iii^	71.14 (9)	O3—K1—Mo1^xi^	115.47 (4)
O4—Mo1—K1^x^	36.07 (8)	O3^xii^—K1—Mo1^xii^	28.44 (4)
O4—Mo1—K1	41.90 (7)	O3—K1—Mo1^xii^	52.19 (4)
O4—Mo1—O2	109.34 (11)	O3—K1—O3^xii^	48.36 (8)
O4—Mo1—O1	106.78 (11)	Tb1^iv^—O2—Tb1^xvi^	109.07 (9)
O4—Mo1—O3	105.28 (10)	Mo1—O2—Tb1^iv^	127.76 (10)
O1—Mo1—K1	121.85 (7)	Mo1—O2—Tb1^xvi^	120.07 (9)
O1—Mo1—K1^iii^	43.35 (7)	Mo1—O4—K1	112.78 (11)
O1—Mo1—K1^x^	95.66 (7)	Mo1—O4—K1^x^	122.51 (11)
O1—Mo1—O2	118.69 (10)	K1—O4—K1^x^	122.78 (9)
O3—Mo1—K1	63.88 (7)	Tb1—O1—K1^iii^	112.84 (9)
O3—Mo1—K1^iii^	90.54 (8)	Mo1—O1—Tb1	125.44 (11)
O3—Mo1—K1^x^	141.01 (7)	Mo1—O1—K1^iii^	110.75 (10)
O3—Mo1—O2	111.67 (10)	Tb1^ii^—O3—K1	145.20 (9)
O3—Mo1—O1	104.16 (12)	Mo1—O3—Tb1^ii^	127.02 (11)
Mo1—K1—Mo1^xi^	138.799 (13)	Mo1—O3—K1	87.68 (8)

Symmetry codes: (i) −*x*, −*y*+1, −*z*; (ii) −*x*, −*y*+1, −*z*+1; (iii) −*x*+1/2, −*y*+3/2, *z*−1/2; (iv) −*x*+1, −*y*+1, −*z*+1; (v) *x*−1, −*y*+1, *z*−1/2; (vi) −*x*+1, *y*, −*z*+1/2; (vii) *x*−1, *y*, *z*; (viii) −*x*, *y*, −*z*+1/2; (ix) *x*, −*y*+1, *z*−1/2; (x) −*x*+3/2, −*y*+3/2, *z*−1/2; (xi) −*x*+1/2, −*y*+3/2, *z*+1/2; (xii) −*x*+1, *y*, −*z*+3/2; (xiii) *x*+1/2, −*y*+3/2, −*z*+1; (xiv) *x*−1/2, −*y*+3/2, −*z*+1; (xv) −*x*+3/2, −*y*+3/2, *z*+1/2; (xvi) *x*+1, *y*, *z*.

### Potassium dysprosium bis(molybdate) (Dy_Molybdate) Crystal data

**Table:**

KDy(MoO_4_)_2_	*F*(000) = 932
*M**_r_* = 521.48	*D*_x_ = 4.739 Mg m^−^^3^
Orthorhombic, *P**b**c**n*	Mo *K*α radiation, λ = 0.71073 Å
Hall symbol: -P 2n 2ab	Cell parameters from 5121 reflections
*a* = 5.0776 (2) Å	θ = 2.3–33.3°
*b* = 18.1214 (7) Å	µ = 14.07 mm^−^^1^
*c* = 7.9428 (3) Å	*T* = 100 K
*V* = 730.84 (5) Å^3^	Plate, light yellow
*Z* = 4	0.11 × 0.11 × 0.03 mm

### Potassium dysprosium bis(molybdate) (Dy_Molybdate) Data collection

**Table:**

Rigaku XtaLAB Synergy-S, HyPix diffractometer	1388 independent reflections
Radiation source: micro-focus sealed X-ray tube	1196 reflections with *I* > 2σ(*I*)
Mirror monochromator	*R*_int_ = 0.075
Detector resolution: 10.0000 pixels mm^-1^	θ_max_ = 34.0°, θ_min_ = 2.3°
ω scans	*h* = −7→7
Absorption correction: gaussian (CrysAlisPro; Rigaku OD, 2019)	*k* = −27→27
*T*_min_ = 0.247, *T*_max_ = 0.695	*l* = −11→11
17317 measured reflections	

### Potassium dysprosium bis(molybdate) (Dy_Molybdate) Refinement

**Table:**

Refinement on *F*^2^	0 restraints
Least-squares matrix: full	0 constraints
*R*[*F*^2^ > 2σ(*F*^2^)] = 0.033	Primary atom site location: dual
*wR*(*F*^2^) = 0.088	*w* = 1/[σ^2^(*F*_o_^2^) + (0.0343*P*)^2^ + 8.538*P*] where *P* = (*F*_o_^2^ + 2*F*_c_^2^)/3
*S* = 1.10	(Δ/σ)_max_ = 0.001
1388 reflections	Δρ_max_ = 2.38 e Å^−^^3^
56 parameters	Δρ_min_ = −2.65 e Å^−^^3^

### Potassium dysprosium bis(molybdate) (Dy_Molybdate) Special details

**Table:**

Geometry. All esds (except the esd in the dihedral angle between two l.s. planes) are estimated using the full covariance matrix. The cell esds are taken into account individually in the estimation of esds in distances, angles and torsion angles; correlations between esds in cell parameters are only used when they are defined by crystal symmetry. An approximate (isotropic) treatment of cell esds is used for estimating esds involving l.s. planes.
Refinement. Reflections were merged by SHELXL according to the crystal class for the calculation of statistics and refinement. _reflns_Friedel_fraction is defined as the number of unique Friedel pairs measured divided by the number that would be possible theoretically, ignoring centric projections and systematic absences.

### Potassium dysprosium bis(molybdate) (Dy_Molybdate) Fractional atomic coordinates and isotropic or equivalent isotropic displacement parameters (Å^2^)

**Table:**

	*x*	*y*	*z*	*U*_iso_*/*U*_eq_	
Dy1	0.000000	0.49408 (2)	0.250000	0.00749 (11)	
Mo1	0.48037 (7)	0.60238 (2)	0.48347 (5)	0.00769 (11)	
K1	0.500000	0.77076 (9)	0.750000	0.0118 (3)	
O4	0.6101 (7)	0.6900 (2)	0.4770 (4)	0.0121 (6)	
O2	0.7489 (7)	0.53449 (19)	0.5049 (4)	0.0100 (6)	
O1	0.2588 (6)	0.59449 (19)	0.3083 (4)	0.0111 (6)	
O3	0.2709 (6)	0.60172 (19)	0.6610 (4)	0.0109 (6)	

### Potassium dysprosium bis(molybdate) (Dy_Molybdate) Atomic displacement parameters (Å^2^)

**Table:**

	*U*^11^	*U*^22^	*U*^33^	*U*^12^	*U*^13^	*U*^23^
Dy1	0.00510 (15)	0.01210 (17)	0.00526 (16)	0.000	−0.00001 (8)	0.000
Mo1	0.00512 (18)	0.0115 (2)	0.00648 (18)	0.00032 (12)	−0.00022 (10)	−0.00023 (12)
K1	0.0125 (6)	0.0147 (6)	0.0083 (6)	0.000	−0.0006 (4)	0.000
O4	0.0098 (15)	0.0154 (16)	0.0109 (15)	−0.0020 (12)	0.0006 (11)	−0.0004 (12)
O2	0.0066 (13)	0.0161 (16)	0.0072 (14)	0.0018 (11)	−0.0009 (11)	−0.0015 (11)
O1	0.0081 (13)	0.0178 (16)	0.0074 (14)	−0.0013 (12)	−0.0010 (11)	0.0001 (12)
O3	0.0073 (13)	0.0171 (16)	0.0082 (14)	−0.0003 (12)	0.0008 (11)	0.0006 (11)

### Potassium dysprosium bis(molybdate) (Dy_Molybdate) Geometric parameters (Å, º)

**Table:**

Dy1—Dy1^i^	3.9772 (1)	Mo1—O3	1.766 (3)
Dy1—Dy1^ii^	3.9772 (2)	K1—Mo1^xi^	3.8305 (10)
Dy1—K1^iii^	4.2614 (16)	K1—Mo1^xii^	3.7150 (13)
Dy1—O2^iv^	2.384 (3)	K1—Mo1^xiii^	3.8305 (10)
Dy1—O2^v^	2.384 (3)	K1—O4	2.676 (4)
Dy1—O2^vi^	2.502 (3)	K1—O4^xiv^	2.770 (3)
Dy1—O2^vii^	2.502 (3)	K1—O4^xii^	2.676 (4)
Dy1—O1	2.292 (3)	K1—O4^xv^	2.770 (3)
Dy1—O1^viii^	2.292 (3)	K1—O1^xiii^	2.812 (4)
Dy1—O3^ix^	2.325 (3)	K1—O1^xi^	2.812 (4)
Dy1—O3^ii^	2.325 (3)	K1—O3^xii^	3.352 (4)
Mo1—K1	3.7150 (13)	K1—O3	3.352 (4)
Mo1—K1^x^	3.9604 (10)	O4—K1^x^	2.770 (3)
Mo1—K1^iii^	3.8305 (10)	O2—Dy1^xvi^	2.502 (3)
Mo1—O4	1.720 (4)	O2—Dy1^iv^	2.384 (3)
Mo1—O2	1.845 (3)	O1—K1^iii^	2.812 (4)
Mo1—O1	1.795 (3)	O3—Dy1^ii^	2.325 (3)
			
Dy1^ii^—Dy1—Dy1^i^	173.818 (18)	Mo1—K1—Mo1^xii^	69.57 (3)
Dy1^ii^—Dy1—K1^iii^	86.909 (9)	Mo1—K1—Mo1^xi^	138.737 (15)
Dy1^i^—Dy1—K1^iii^	86.909 (9)	Mo1^xii^—K1—Mo1^xiii^	138.737 (15)
O2^iv^—Dy1—Dy1^i^	145.80 (8)	Mo1^xiii^—K1—Mo1^xi^	106.24 (4)
O2^v^—Dy1—Dy1^i^	36.52 (8)	Mo1^xii^—K1—Mo1^xi^	103.540 (12)
O2^vi^—Dy1—Dy1^i^	34.53 (8)	O4^xv^—K1—Mo1^xii^	79.68 (8)
O2^vii^—Dy1—Dy1^i^	142.40 (8)	O4—K1—Mo1^xii^	90.40 (9)
O2^iv^—Dy1—Dy1^ii^	36.52 (8)	O4^xiv^—K1—Mo1^xi^	72.90 (7)
O2^v^—Dy1—Dy1^ii^	145.80 (8)	O4^xii^—K1—Mo1	90.40 (9)
O2^vi^—Dy1—Dy1^ii^	142.40 (8)	O4^xii^—K1—Mo1^xi^	78.63 (8)
O2^vii^—Dy1—Dy1^ii^	34.53 (8)	O4^xv^—K1—Mo1	126.93 (8)
O2^vi^—Dy1—K1^iii^	72.98 (8)	O4^xiv^—K1—Mo1	79.68 (8)
O2^vii^—Dy1—K1^iii^	72.98 (8)	O4—K1—Mo1^xiii^	78.63 (8)
O2^v^—Dy1—K1^iii^	102.55 (8)	O4—K1—Mo1^xi^	148.63 (8)
O2^iv^—Dy1—K1^iii^	102.55 (8)	O4^xii^—K1—Mo1^xiii^	148.63 (8)
O2^vii^—Dy1—O2^vi^	145.97 (16)	O4^xv^—K1—Mo1^xi^	89.19 (8)
O2^iv^—Dy1—O2^vi^	116.87 (14)	O4^xv^—K1—Mo1^xiii^	72.90 (7)
O2^iv^—Dy1—O2^vii^	71.05 (13)	O4—K1—Mo1	25.09 (8)
O2^v^—Dy1—O2^vi^	71.05 (13)	O4^xiv^—K1—Mo1^xiii^	89.19 (8)
O2^v^—Dy1—O2^vii^	116.87 (14)	O4^xii^—K1—Mo1^xii^	25.09 (8)
O2^v^—Dy1—O2^iv^	154.91 (17)	O4^xiv^—K1—Mo1^xii^	126.93 (8)
O1^viii^—Dy1—Dy1^i^	75.84 (8)	O4^xii^—K1—O4^xv^	76.26 (9)
O1—Dy1—Dy1^i^	99.15 (8)	O4—K1—O4^xv^	121.24 (5)
O1^viii^—Dy1—Dy1^ii^	99.15 (8)	O4^xv^—K1—O4^xiv^	150.24 (16)
O1—Dy1—Dy1^ii^	75.84 (8)	O4—K1—O4^xiv^	76.25 (8)
O1—Dy1—K1^iii^	37.45 (8)	O4^xii^—K1—O4^xiv^	121.24 (5)
O1^viii^—Dy1—K1^iii^	37.45 (8)	O4—K1—O4^xii^	113.67 (16)
O1—Dy1—O2^iv^	72.59 (12)	O4^xii^—K1—O1^xi^	104.12 (10)
O1^viii^—Dy1—O2^vi^	84.04 (12)	O4—K1—O1^xi^	134.94 (11)
O1—Dy1—O2^vii^	84.04 (12)	O4—K1—O1^xiii^	104.12 (10)
O1—Dy1—O2^vi^	68.85 (11)	O4^xiv^—K1—O1^xiii^	90.21 (11)
O1^viii^—Dy1—O2^iv^	130.11 (12)	O4^xiv^—K1—O1^xi^	63.27 (10)
O1^viii^—Dy1—O2^vii^	68.85 (11)	O4^xii^—K1—O1^xiii^	134.94 (11)
O1—Dy1—O2^v^	130.11 (12)	O4^xv^—K1—O1^xiii^	63.27 (10)
O1^viii^—Dy1—O2^v^	72.59 (12)	O4^xv^—K1—O1^xi^	90.21 (11)
O1^viii^—Dy1—O1	74.89 (17)	O4^xv^—K1—O3^xii^	81.35 (10)
O1—Dy1—O3^ix^	108.36 (12)	O4^xv^—K1—O3	128.30 (10)
O1—Dy1—O3^ii^	150.54 (12)	O4^xii^—K1—O3^xii^	53.25 (10)
O1^viii^—Dy1—O3^ix^	150.54 (12)	O4^xiv^—K1—O3^xii^	128.30 (10)
O1^viii^—Dy1—O3^ii^	108.36 (12)	O4^xiv^—K1—O3	81.35 (10)
O3^ix^—Dy1—Dy1^i^	74.74 (8)	O4—K1—O3	53.25 (10)
O3^ix^—Dy1—Dy1^ii^	110.11 (8)	O4—K1—O3^xii^	66.32 (10)
O3^ii^—Dy1—Dy1^ii^	74.74 (8)	O4^xii^—K1—O3	66.32 (10)
O3^ii^—Dy1—Dy1^i^	110.11 (8)	O1^xiii^—K1—Mo1	129.20 (7)
O3^ii^—Dy1—K1^iii^	138.31 (8)	O1^xiii^—K1—Mo1^xi^	81.73 (8)
O3^ix^—Dy1—K1^iii^	138.31 (8)	O1^xi^—K1—Mo1^xii^	129.20 (7)
O3^ix^—Dy1—O2^vii^	139.98 (11)	O1^xi^—K1—Mo1	142.63 (7)
O3^ix^—Dy1—O2^iv^	76.68 (11)	O1^xiii^—K1—Mo1^xii^	142.63 (7)
O3^ii^—Dy1—O2^vii^	70.80 (12)	O1^xiii^—K1—Mo1^xiii^	26.02 (7)
O3^ii^—Dy1—O2^v^	76.68 (11)	O1^xi^—K1—Mo1^xiii^	81.73 (8)
O3^ix^—Dy1—O2^v^	84.61 (12)	O1^xi^—K1—Mo1^xi^	26.02 (7)
O3^ix^—Dy1—O2^vi^	70.80 (12)	O1^xiii^—K1—O1^xi^	59.42 (14)
O3^ii^—Dy1—O2^iv^	84.61 (12)	O1^xiii^—K1—O3	157.10 (9)
O3^ii^—Dy1—O2^vi^	139.98 (11)	O1^xi^—K1—O3^xii^	157.10 (9)
O3^ii^—Dy1—O3^ix^	83.39 (16)	O1^xi^—K1—O3	131.78 (9)
K1—Mo1—K1^iii^	78.467 (11)	O1^xiii^—K1—O3^xii^	131.78 (9)
K1—Mo1—K1^x^	76.833 (11)	O3^xii^—K1—Mo1^xiii^	115.44 (6)
K1^iii^—Mo1—K1^x^	81.33 (3)	O3^xii^—K1—Mo1	51.60 (6)
O4—Mo1—K1^x^	36.37 (12)	O3—K1—Mo1^xi^	115.44 (6)
O4—Mo1—K1	41.29 (11)	O3—K1—Mo1^xiii^	131.86 (6)
O4—Mo1—K1^iii^	71.06 (12)	O3—K1—Mo1	28.36 (6)
O4—Mo1—O2	109.59 (16)	O3^xii^—K1—Mo1^xi^	131.86 (6)
O4—Mo1—O1	106.89 (16)	O3—K1—Mo1^xii^	51.60 (6)
O4—Mo1—O3	105.13 (16)	O3^xii^—K1—Mo1^xii^	28.36 (6)
O2—Mo1—K1^x^	86.44 (11)	O3^xii^—K1—O3	47.91 (11)
O2—Mo1—K1^iii^	156.32 (9)	Mo1—O4—K1^x^	122.04 (17)
O2—Mo1—K1	118.38 (10)	Mo1—O4—K1	113.61 (16)
O1—Mo1—K1^iii^	43.41 (11)	K1—O4—K1^x^	122.44 (13)
O1—Mo1—K1	121.60 (11)	Dy1^iv^—O2—Dy1^xvi^	108.95 (13)
O1—Mo1—K1^x^	95.79 (11)	Mo1—O2—Dy1^xvi^	119.80 (14)
O1—Mo1—O2	118.81 (15)	Mo1—O2—Dy1^iv^	128.01 (15)
O3—Mo1—K1^iii^	90.42 (11)	Dy1—O1—K1^iii^	112.84 (12)
O3—Mo1—K1^x^	141.16 (11)	Mo1—O1—Dy1	125.41 (17)
O3—Mo1—K1	64.32 (11)	Mo1—O1—K1^iii^	110.57 (15)
O3—Mo1—O2	111.51 (15)	Dy1^ii^—O3—K1	145.45 (13)
O3—Mo1—O1	103.94 (16)	Mo1—O3—Dy1^ii^	127.15 (18)
Mo1—K1—Mo1^xiii^	103.540 (12)	Mo1—O3—K1	87.32 (12)

Symmetry codes: (i) −*x*, −*y*+1, −*z*; (ii) −*x*, −*y*+1, −*z*+1; (iii) −*x*+1/2, −*y*+3/2, *z*−1/2; (iv) −*x*+1, −*y*+1, −*z*+1; (v) *x*−1, −*y*+1, *z*−1/2; (vi) −*x*+1, *y*, −*z*+1/2; (vii) *x*−1, *y*, *z*; (viii) −*x*, *y*, −*z*+1/2; (ix) *x*, −*y*+1, *z*−1/2; (x) −*x*+3/2, −*y*+3/2, *z*−1/2; (xi) −*x*+1/2, −*y*+3/2, *z*+1/2; (xii) −*x*+1, *y*, −*z*+3/2; (xiii) *x*+1/2, −*y*+3/2, −*z*+1; (xiv) *x*−1/2, −*y*+3/2, −*z*+1; (xv) −*x*+3/2, −*y*+3/2, *z*+1/2; (xvi) *x*+1, *y*, *z*.

### Potassium holmium bis(molybdate) (Ho_Molybdate) Crystal data

**Table:**

KHo(MoO_4_)_2_	*F*(000) = 936
*M**_r_* = 523.91	*D*_x_ = 4.757 Mg m^−^^3^
Orthorhombic, *P**b**c**n*	Mo *K*α radiation, λ = 0.71073 Å
Hall symbol: -P 2n 2ab	Cell parameters from 2917 reflections
*a* = 5.0770 (15) Å	θ = 3.4–28.8°
*b* = 18.161 (5) Å	µ = 14.66 mm^−^^1^
*c* = 7.934 (2) Å	*T* = 273 K
*V* = 731.5 (4) Å^3^	Plate, light red
*Z* = 4	0.06 × 0.06 × 0.04 mm

### Potassium holmium bis(molybdate) (Ho_Molybdate) Data collection

**Table:**

Bruker APEXII CCD diffractometer	818 reflections with *I* > 2σ(*I*)
Radiation source: X-ray tube	*R*_int_ = 0.032
φ and ω scans	θ_max_ = 28.9°, θ_min_ = 2.2°
Absorption correction: multi-scan (SADABS; Krause et al., 2015)	*h* = −6→6
*T*_min_ = 0.248, *T*_max_ = 0.343	*k* = −24→24
7665 measured reflections	*l* = −10→10
936 independent reflections	

### Potassium holmium bis(molybdate) (Ho_Molybdate) Refinement

**Table:**

Refinement on *F*^2^	0 restraints
Least-squares matrix: full	0 constraints
*R*[*F*^2^ > 2σ(*F*^2^)] = 0.027	Primary atom site location: dual
*wR*(*F*^2^) = 0.063	*w* = 1/[σ^2^(*F*_o_^2^) + 11.609*P*] where *P* = (*F*_o_^2^ + 2*F*_c_^2^)/3
*S* = 1.25	(Δ/σ)_max_ = 0.001
936 reflections	Δρ_max_ = 1.51 e Å^−^^3^
56 parameters	Δρ_min_ = −1.37 e Å^−^^3^

### Potassium holmium bis(molybdate) (Ho_Molybdate) Special details

**Table:**

Geometry. All esds (except the esd in the dihedral angle between two l.s. planes) are estimated using the full covariance matrix. The cell esds are taken into account individually in the estimation of esds in distances, angles and torsion angles; correlations between esds in cell parameters are only used when they are defined by crystal symmetry. An approximate (isotropic) treatment of cell esds is used for estimating esds involving l.s. planes.
Refinement. Reflections were merged by SHELXL according to the crystal class for the calculation of statistics and refinement. _reflns_Friedel_fraction is defined as the number of unique Friedel pairs measured divided by the number that would be possible theoretically, ignoring centric projections and systematic absences.

### Potassium holmium bis(molybdate) (Ho_Molybdate) Fractional atomic coordinates and isotropic or equivalent isotropic displacement parameters (Å^2^)

**Table:**

	*x*	*y*	*z*	*U*_iso_*/*U*_eq_	
Ho1	0.000000	0.49423 (2)	0.750000	0.00704 (12)	
Mo1	0.47909 (9)	0.60179 (3)	0.51614 (6)	0.00726 (13)	
K1	0.500000	0.77102 (11)	0.250000	0.0141 (4)	
O4	0.6095 (9)	0.6894 (3)	0.5227 (6)	0.0122 (9)	
O2	0.7503 (9)	0.5342 (2)	0.4941 (5)	0.0095 (8)	
O1	0.2583 (8)	0.5939 (2)	0.6923 (5)	0.0096 (8)	
O3	0.2700 (9)	0.6008 (2)	0.3389 (5)	0.0093 (8)	

### Potassium holmium bis(molybdate) (Ho_Molybdate) Atomic displacement parameters (Å^2^)

**Table:**

	*U*^11^	*U*^22^	*U*^33^	*U*^12^	*U*^13^	*U*^23^
Ho1	0.00566 (18)	0.0094 (2)	0.00606 (18)	0.000	−0.00040 (14)	0.000
Mo1	0.0058 (2)	0.0084 (3)	0.0076 (2)	0.00040 (19)	−0.00009 (17)	0.00032 (17)
K1	0.0177 (9)	0.0132 (9)	0.0115 (8)	0.000	0.0013 (8)	0.000
O4	0.013 (2)	0.014 (2)	0.010 (2)	−0.0011 (18)	0.0002 (17)	0.0016 (17)
O2	0.0080 (19)	0.014 (2)	0.0061 (19)	0.0016 (17)	0.0037 (18)	−0.0005 (17)
O1	0.0072 (19)	0.013 (2)	0.0085 (19)	−0.0001 (17)	0.0015 (16)	0.0001 (17)
O3	0.013 (2)	0.012 (2)	0.0035 (19)	−0.0008 (19)	−0.0005 (15)	0.0000 (16)

### Potassium holmium bis(molybdate) (Ho_Molybdate) Geometric parameters (Å, º)

**Table:**

Ho1—Ho1^i^	3.9723 (11)	Mo1—O3	1.762 (4)
Ho1—Ho1^ii^	3.9723 (11)	K1—Mo1^xi^	3.8332 (14)
Ho1—K1^iii^	4.263 (2)	K1—Mo1^xii^	3.7303 (18)
Ho1—O2^iv^	2.372 (4)	K1—Mo1^xiii^	3.8332 (14)
Ho1—O2^v^	2.372 (4)	K1—O4	2.680 (5)
Ho1—O2^vi^	2.501 (4)	K1—O4^xiv^	2.775 (5)
Ho1—O2^vii^	2.501 (4)	K1—O4^xii^	2.681 (5)
Ho1—O1^viii^	2.282 (4)	K1—O4^xv^	2.775 (5)
Ho1—O1	2.282 (4)	K1—O1^xiii^	2.819 (5)
Ho1—O3^ix^	2.314 (4)	K1—O1^xi^	2.819 (5)
Ho1—O3^i^	2.314 (4)	K1—O3	3.380 (5)
Mo1—K1	3.7303 (18)	K1—O3^xii^	3.380 (5)
Mo1—K1^x^	3.9713 (14)	O4—K1^x^	2.775 (5)
Mo1—K1^iii^	3.8332 (14)	O2—Ho1^xvi^	2.501 (4)
Mo1—O4	1.724 (5)	O2—Ho1^v^	2.372 (4)
Mo1—O2	1.853 (4)	O1—K1^iii^	2.819 (5)
Mo1—O1	1.797 (4)	O3—Ho1^i^	2.314 (4)
			
Ho1^ii^—Ho1—Ho1^i^	173.96 (2)	Mo1^xii^—K1—Mo1	69.05 (4)
Ho1^ii^—Ho1—K1^iii^	86.979 (11)	Mo1^xii^—K1—Mo1^xi^	103.92 (2)
Ho1^i^—Ho1—K1^iii^	86.979 (11)	Mo1—K1—Mo1^xiii^	103.92 (2)
O2^iv^—Ho1—Ho1^i^	145.79 (11)	Mo1^xiii^—K1—Mo1^xi^	105.89 (5)
O2^v^—Ho1—Ho1^i^	36.49 (11)	Mo1—K1—Mo1^xi^	138.79 (2)
O2^vi^—Ho1—Ho1^i^	34.33 (10)	O4^xv^—K1—Mo1	126.98 (10)
O2^vii^—Ho1—Ho1^i^	142.68 (10)	O4—K1—Mo1	24.98 (10)
O2^iv^—Ho1—Ho1^ii^	36.49 (11)	O4^xiv^—K1—Mo1^xi^	72.85 (10)
O2^v^—Ho1—Ho1^ii^	145.79 (11)	O4^xii^—K1—Mo1^xii^	24.98 (10)
O2^vi^—Ho1—Ho1^ii^	142.68 (10)	O4^xii^—K1—Mo1^xi^	79.11 (10)
O2^vii^—Ho1—Ho1^ii^	34.33 (10)	O4^xv^—K1—Mo1^xii^	79.93 (10)
O2^vi^—Ho1—K1^iii^	73.13 (10)	O4^xiv^—K1—Mo1^xii^	126.98 (10)
O2^vii^—Ho1—K1^iii^	73.13 (10)	O4—K1—Mo1^xiii^	79.11 (10)
O2^v^—Ho1—K1^iii^	102.57 (11)	O4—K1—Mo1^xi^	148.80 (10)
O2^iv^—Ho1—K1^iii^	102.57 (11)	O4^xii^—K1—Mo1^xiii^	148.80 (10)
O2^vii^—Ho1—O2^vi^	146.3 (2)	O4^xv^—K1—Mo1^xi^	89.01 (10)
O2^iv^—Ho1—O2^vi^	117.06 (18)	O4^xv^—K1—Mo1^xiii^	72.85 (10)
O2^iv^—Ho1—O2^vii^	70.81 (17)	O4—K1—Mo1^xii^	89.73 (11)
O2^v^—Ho1—O2^vi^	70.81 (17)	O4^xiv^—K1—Mo1^xiii^	89.01 (10)
O2^v^—Ho1—O2^vii^	117.06 (18)	O4^xii^—K1—Mo1	89.73 (11)
O2^v^—Ho1—O2^iv^	154.9 (2)	O4^xiv^—K1—Mo1	79.93 (10)
O1—Ho1—Ho1^i^	75.99 (11)	O4^xii^—K1—O4^xv^	76.52 (11)
O1^viii^—Ho1—Ho1^i^	99.12 (11)	O4—K1—O4^xv^	121.30 (6)
O1—Ho1—Ho1^ii^	99.12 (11)	O4^xv^—K1—O4^xiv^	150.0 (2)
O1^viii^—Ho1—Ho1^ii^	75.99 (11)	O4—K1—O4^xiv^	76.52 (11)
O1^viii^—Ho1—K1^iii^	37.49 (11)	O4^xii^—K1—O4^xiv^	121.30 (6)
O1—Ho1—K1^iii^	37.50 (11)	O4—K1—O4^xii^	112.9 (2)
O1^viii^—Ho1—O2^iv^	72.64 (15)	O4^xii^—K1—O1^xi^	104.69 (13)
O1—Ho1—O2^vi^	84.17 (15)	O4—K1—O1^xi^	135.12 (13)
O1^viii^—Ho1—O2^vii^	84.17 (15)	O4—K1—O1^xiii^	104.69 (13)
O1^viii^—Ho1—O2^vi^	68.97 (15)	O4^xiv^—K1—O1^xiii^	90.09 (14)
O1—Ho1—O2^iv^	130.07 (15)	O4^xiv^—K1—O1^xi^	63.11 (13)
O1—Ho1—O2^vii^	68.97 (15)	O4^xii^—K1—O1^xiii^	135.13 (13)
O1^viii^—Ho1—O2^v^	130.06 (15)	O4^xv^—K1—O1^xiii^	63.11 (13)
O1—Ho1—O2^v^	72.64 (15)	O4^xv^—K1—O1^xi^	90.09 (14)
O1—Ho1—O1^viii^	75.0 (2)	O4^xv^—K1—O3	128.28 (13)
O1^viii^—Ho1—O3^ix^	150.57 (15)	O4^xv^—K1—O3^xii^	81.63 (12)
O1^viii^—Ho1—O3^i^	108.19 (16)	O4^xii^—K1—O3	65.85 (13)
O1—Ho1—O3^ix^	108.19 (16)	O4^xiv^—K1—O3	81.63 (12)
O1—Ho1—O3^i^	150.57 (15)	O4^xiv^—K1—O3^xii^	128.28 (13)
O3^ix^—Ho1—Ho1^i^	110.11 (10)	O4—K1—O3^xii^	65.85 (13)
O3^ix^—Ho1—Ho1^ii^	74.62 (10)	O4—K1—O3	52.94 (12)
O3^i^—Ho1—Ho1^ii^	110.11 (10)	O4^xii^—K1—O3^xii^	52.93 (12)
O3^i^—Ho1—Ho1^i^	74.62 (10)	O1^xiii^—K1—Mo1^xii^	142.71 (9)
O3^i^—Ho1—K1^iii^	138.22 (11)	O1^xiii^—K1—Mo1^xi^	81.34 (10)
O3^ix^—Ho1—K1^iii^	138.21 (11)	O1^xi^—K1—Mo1	142.71 (9)
O3^ix^—Ho1—O2^vii^	70.64 (15)	O1^xi^—K1—Mo1^xii^	129.66 (9)
O3^ix^—Ho1—O2^iv^	84.58 (15)	O1^xiii^—K1—Mo1	129.66 (9)
O3^i^—Ho1—O2^vii^	139.83 (14)	O1^xiii^—K1—Mo1^xiii^	26.09 (9)
O3^i^—Ho1—O2^v^	84.58 (15)	O1^xi^—K1—Mo1^xiii^	81.34 (10)
O3^ix^—Ho1—O2^v^	76.70 (15)	O1^xi^—K1—Mo1^xi^	26.09 (9)
O3^ix^—Ho1—O2^vi^	139.83 (14)	O1^xiii^—K1—O1^xi^	59.05 (18)
O3^i^—Ho1—O2^iv^	76.70 (15)	O1^xiii^—K1—O3^xii^	132.01 (12)
O3^i^—Ho1—O2^vi^	70.64 (15)	O1^xi^—K1—O3	132.01 (12)
O3^i^—Ho1—O3^ix^	83.6 (2)	O1^xi^—K1—O3^xii^	157.36 (11)
K1—Mo1—K1^iii^	78.20 (2)	O1^xiii^—K1—O3	157.36 (11)
K1—Mo1—K1^x^	76.49 (2)	O3—K1—Mo1^xiii^	132.03 (7)
K1^iii^—Mo1—K1^x^	81.14 (4)	O3—K1—Mo1^xii^	51.26 (8)
O4—Mo1—K1^x^	36.25 (15)	O3^xii^—K1—Mo1^xi^	132.03 (7)
O4—Mo1—K1	41.04 (15)	O3^xii^—K1—Mo1^xiii^	115.66 (8)
O4—Mo1—K1^iii^	70.92 (15)	O3^xii^—K1—Mo1^xii^	28.14 (7)
O4—Mo1—O2	109.2 (2)	O3—K1—Mo1^xi^	115.66 (8)
O4—Mo1—O1	106.8 (2)	O3^xii^—K1—Mo1	51.26 (8)
O4—Mo1—O3	105.4 (2)	O3—K1—Mo1	28.14 (7)
O2—Mo1—K1^x^	86.25 (14)	O3—K1—O3^xii^	47.61 (15)
O2—Mo1—K1^iii^	156.40 (13)	Mo1—O4—K1^x^	122.2 (2)
O2—Mo1—K1	118.13 (13)	Mo1—O4—K1	114.0 (2)
O1—Mo1—K1^iii^	43.60 (14)	K1—O4—K1^x^	121.97 (17)
O1—Mo1—K1	121.56 (14)	Ho1^v^—O2—Ho1^xvi^	109.19 (17)
O1—Mo1—K1^x^	95.64 (14)	Mo1—O2—Ho1^xvi^	119.50 (19)
O1—Mo1—O2	118.95 (19)	Mo1—O2—Ho1^v^	128.2 (2)
O3—Mo1—K1^iii^	90.59 (14)	Ho1—O1—K1^iii^	112.98 (16)
O3—Mo1—K1^x^	141.28 (15)	Mo1—O1—Ho1	125.3 (2)
O3—Mo1—K1	64.79 (14)	Mo1—O1—K1^iii^	110.32 (19)
O3—Mo1—O2	111.44 (19)	Ho1^i^—O3—K1	145.41 (16)
O3—Mo1—O1	104.1 (2)	Mo1—O3—Ho1^i^	127.5 (2)
Mo1^xii^—K1—Mo1^xiii^	138.79 (2)	Mo1—O3—K1	87.06 (16)

Symmetry codes: (i) −*x*, −*y*+1, −*z*+1; (ii) −*x*, −*y*+1, −*z*+2; (iii) −*x*+1/2, −*y*+3/2, *z*+1/2; (iv) *x*−1, −*y*+1, *z*+1/2; (v) −*x*+1, −*y*+1, −*z*+1; (vi) *x*−1, *y*, *z*; (vii) −*x*+1, *y*, −*z*+3/2; (viii) −*x*, *y*, −*z*+3/2; (ix) *x*, −*y*+1, *z*+1/2; (x) −*x*+3/2, −*y*+3/2, *z*+1/2; (xi) −*x*+1/2, −*y*+3/2, *z*−1/2; (xii) −*x*+1, *y*, −*z*+1/2; (xiii) *x*+1/2, −*y*+3/2, −*z*+1; (xiv) *x*−1/2, −*y*+3/2, −*z*+1; (xv) −*x*+3/2, −*y*+3/2, *z*−1/2; (xvi) *x*+1, *y*, *z*.

### Potassium erbium bis(molybdate) (Er_Molybdate) Crystal data

**Table:**

KEr(MoO_4_)_2_	*F*(000) = 940
*M**_r_* = 526.24	*D*_x_ = 4.810 Mg m^−^^3^
Orthorhombic, *P**b**c**n*	Mo *K*α radiation, λ = 0.71073 Å
Hall symbol: -P 2n 2ab	Cell parameters from 7328 reflections
*a* = 5.0602 (2) Å	θ = 3.4–34.0°
*b* = 18.1965 (8) Å	µ = 15.42 mm^−^^1^
*c* = 7.8920 (3) Å	*T* = 100 K
*V* = 726.68 (5) Å^3^	Plate, light pink
*Z* = 4	0.28 × 0.15 × 0.02 mm

### Potassium erbium bis(molybdate) (Er_Molybdate) Data collection

**Table:**

Rigaku XtaLAB Synergy-S, HyPix diffractometer	1386 independent reflections
Radiation source: micro-focus sealed X-ray tube	1255 reflections with *I* > 2σ(*I*)
Mirror monochromator	*R*_int_ = 0.072
Detector resolution: 10.0000 pixels mm^-1^	θ_max_ = 34.0°, θ_min_ = 2.2°
ω scans	*h* = −7→7
Absorption correction: gaussian (CrysAlisPro; Rigaku OD, 2019)	*k* = −28→27
*T*_min_ = 0.091, *T*_max_ = 0.911	*l* = −12→10
21246 measured reflections	

### Potassium erbium bis(molybdate) (Er_Molybdate) Refinement

**Table:**

Refinement on *F*^2^	0 restraints
Least-squares matrix: full	0 constraints
*R*[*F*^2^ > 2σ(*F*^2^)] = 0.027	Primary atom site location: dual
*wR*(*F*^2^) = 0.073	*w* = 1/[σ^2^(*F*_o_^2^) + (0.0345*P*)^2^ + 4.1539*P*] where *P* = (*F*_o_^2^ + 2*F*_c_^2^)/3
*S* = 1.06	(Δ/σ)_max_ = 0.002
1386 reflections	Δρ_max_ = 2.04 e Å^−^^3^
56 parameters	Δρ_min_ = −1.62 e Å^−^^3^

### Potassium erbium bis(molybdate) (Er_Molybdate) Special details

**Table:**

Geometry. All esds (except the esd in the dihedral angle between two l.s. planes) are estimated using the full covariance matrix. The cell esds are taken into account individually in the estimation of esds in distances, angles and torsion angles; correlations between esds in cell parameters are only used when they are defined by crystal symmetry. An approximate (isotropic) treatment of cell esds is used for estimating esds involving l.s. planes.

### Potassium erbium bis(molybdate) (Er_Molybdate) Fractional atomic coordinates and isotropic or equivalent isotropic displacement parameters (Å^2^)

**Table:**

	*x*	*y*	*z*	*U*_iso_*/*U*_eq_	
Er1	1.000000	0.50535 (2)	0.750000	0.00602 (9)	
Mo1	0.52026 (6)	0.39859 (2)	0.51517 (4)	0.00637 (9)	
K1	0.500000	0.22821 (7)	0.250000	0.0107 (2)	
O4	0.3903 (5)	0.31111 (15)	0.5213 (3)	0.0102 (5)	
O2	0.2497 (5)	0.46619 (15)	0.4954 (3)	0.0084 (5)	
O1	0.7417 (5)	0.40581 (14)	0.6914 (3)	0.0100 (4)	
O3	0.7315 (5)	0.39970 (14)	0.3369 (3)	0.0099 (5)	

### Potassium erbium bis(molybdate) (Er_Molybdate) Atomic displacement parameters (Å^2^)

**Table:**

	*U*^11^	*U*^22^	*U*^33^	*U*^12^	*U*^13^	*U*^23^
Er1	0.00367 (12)	0.00995 (13)	0.00443 (13)	0.000	−0.00030 (6)	0.000
Mo1	0.00374 (14)	0.00961 (16)	0.00575 (15)	0.00027 (9)	−0.00044 (8)	−0.00032 (9)
K1	0.0112 (5)	0.0124 (5)	0.0085 (5)	0.000	−0.0007 (3)	0.000
O4	0.0079 (11)	0.0127 (12)	0.0100 (11)	−0.0029 (9)	0.0009 (9)	−0.0003 (9)
O2	0.0052 (10)	0.0131 (12)	0.0067 (10)	0.0007 (9)	−0.0008 (8)	−0.0006 (8)
O1	0.0077 (10)	0.0142 (12)	0.0080 (10)	−0.0014 (9)	−0.0019 (9)	−0.0004 (9)
O3	0.0068 (10)	0.0153 (12)	0.0074 (10)	−0.0014 (9)	0.0006 (8)	−0.0003 (8)

### Potassium erbium bis(molybdate) (Er_Molybdate) Geometric parameters (Å, º)

**Table:**

Er1—Er1^i^	3.9508 (2)	Mo1—O3	1.767 (2)
Er1—Er1^ii^	3.9508 (1)	K1—Mo1^xi^	3.8277 (8)
Er1—K1^iii^	4.2499 (13)	K1—Mo1^xii^	3.7419 (10)
Er1—O2^iv^	2.370 (2)	K1—Mo1^xiii^	3.8277 (8)
Er1—O2^v^	2.370 (2)	K1—O4	2.677 (3)
Er1—O2^vi^	2.478 (2)	K1—O4^xiv^	2.770 (3)
Er1—O2^vii^	2.478 (2)	K1—O4^xii^	2.677 (3)
Er1—O1^viii^	2.281 (2)	K1—O4^xv^	2.770 (3)
Er1—O1	2.281 (3)	K1—O1^xiii^	2.805 (3)
Er1—O3^ix^	2.303 (3)	K1—O1^xi^	2.805 (3)
Er1—O3^ii^	2.303 (3)	K1—O3	3.403 (3)
Mo1—K1	3.7419 (10)	K1—O3^xii^	3.403 (3)
Mo1—K1^x^	3.9610 (8)	O4—K1^x^	2.770 (3)
Mo1—K1^iii^	3.8278 (8)	O2—Er1^xvi^	2.478 (2)
Mo1—O4	1.723 (3)	O2—Er1^iv^	2.370 (2)
Mo1—O2	1.847 (3)	O1—K1^iii^	2.805 (3)
Mo1—O1	1.791 (2)	O3—Er1^ii^	2.303 (3)
			
Er1^ii^—Er1—Er1^i^	174.354 (13)	Mo1^xii^—K1—Mo1	68.10 (2)
Er1^ii^—Er1—K1^iii^	87.177 (7)	Mo1^xii^—K1—Mo1^xi^	104.243 (10)
Er1^i^—Er1—K1^iii^	87.177 (7)	Mo1—K1—Mo1^xiii^	104.243 (10)
O2^iv^—Er1—Er1^i^	145.80 (6)	Mo1^xiii^—K1—Mo1^xi^	105.86 (3)
O2^v^—Er1—Er1^i^	36.34 (6)	Mo1—K1—Mo1^xi^	138.837 (12)
O2^vi^—Er1—Er1^i^	34.52 (6)	O4^xv^—K1—Mo1	126.73 (6)
O2^vii^—Er1—Er1^i^	142.71 (6)	O4—K1—Mo1	24.71 (6)
O2^iv^—Er1—Er1^ii^	36.34 (6)	O4^xiv^—K1—Mo1^xi^	73.00 (5)
O2^v^—Er1—Er1^ii^	145.80 (6)	O4^xii^—K1—Mo1^xii^	24.71 (6)
O2^vi^—Er1—Er1^ii^	142.71 (6)	O4^xii^—K1—Mo1^xi^	79.69 (6)
O2^vii^—Er1—Er1^ii^	34.52 (6)	O4^xv^—K1—Mo1^xii^	80.21 (6)
O2^vi^—Er1—K1^iii^	73.29 (6)	O4^xiv^—K1—Mo1^xii^	126.73 (6)
O2^vii^—Er1—K1^iii^	73.29 (6)	O4—K1—Mo1^xiii^	79.69 (6)
O2^v^—Er1—K1^iii^	102.63 (6)	O4—K1—Mo1^xi^	149.13 (6)
O2^iv^—Er1—K1^iii^	102.63 (6)	O4^xii^—K1—Mo1^xiii^	149.13 (6)
O2^vii^—Er1—O2^vi^	146.57 (12)	O4^xv^—K1—Mo1^xi^	88.91 (6)
O2^iv^—Er1—O2^vi^	116.98 (11)	O4^xv^—K1—Mo1^xiii^	73.00 (5)
O2^iv^—Er1—O2^vii^	70.86 (10)	O4—K1—Mo1^xii^	88.55 (6)
O2^v^—Er1—O2^vi^	70.86 (10)	O4^xiv^—K1—Mo1^xiii^	88.91 (6)
O2^v^—Er1—O2^vii^	116.98 (11)	O4^xii^—K1—Mo1	88.55 (6)
O2^v^—Er1—O2^iv^	154.75 (13)	O4^xiv^—K1—Mo1	80.21 (6)
O1—Er1—Er1^i^	99.40 (6)	O4^xii^—K1—O4^xv^	76.83 (6)
O1^viii^—Er1—Er1^i^	76.03 (6)	O4—K1—O4^xv^	121.26 (4)
O1—Er1—Er1^ii^	76.03 (6)	O4^xv^—K1—O4^xiv^	150.06 (12)
O1^viii^—Er1—Er1^ii^	99.39 (6)	O4—K1—O4^xiv^	76.83 (6)
O1^viii^—Er1—K1^iii^	37.43 (6)	O4^xii^—K1—O4^xiv^	121.26 (4)
O1—Er1—K1^iii^	37.43 (6)	O4—K1—O4^xii^	111.41 (12)
O1^viii^—Er1—O2^iv^	130.14 (9)	O4^xii^—K1—O1^xi^	105.16 (8)
O1—Er1—O2^vi^	69.13 (8)	O4—K1—O1^xi^	135.91 (8)
O1^viii^—Er1—O2^vii^	69.13 (8)	O4—K1—O1^xiii^	105.16 (8)
O1^viii^—Er1—O2^vi^	84.23 (9)	O4^xiv^—K1—O1^xiii^	90.02 (8)
O1—Er1—O2^iv^	72.69 (9)	O4^xiv^—K1—O1^xi^	63.29 (8)
O1—Er1—O2^vii^	84.23 (9)	O4^xii^—K1—O1^xiii^	135.91 (8)
O1^viii^—Er1—O2^v^	72.70 (9)	O4^xv^—K1—O1^xiii^	63.29 (8)
O1—Er1—O2^v^	130.14 (9)	O4^xv^—K1—O1^xi^	90.02 (8)
O1—Er1—O1^viii^	74.85 (13)	O4^xv^—K1—O3	127.86 (8)
O1^viii^—Er1—O3^ix^	150.89 (9)	O4^xv^—K1—O3^xii^	81.96 (7)
O1^viii^—Er1—O3^ii^	108.55 (10)	O4^xii^—K1—O3	64.73 (7)
O1—Er1—O3^ix^	108.55 (10)	O4^xiv^—K1—O3	81.96 (7)
O1—Er1—O3^ii^	150.89 (9)	O4^xiv^—K1—O3^xii^	127.86 (8)
O3^ix^—Er1—Er1^i^	74.90 (6)	O4—K1—O3^xii^	64.73 (7)
O3^ix^—Er1—Er1^ii^	109.54 (6)	O4—K1—O3	52.66 (7)
O3^ii^—Er1—Er1^ii^	74.90 (6)	O4^xii^—K1—O3^xii^	52.66 (7)
O3^ii^—Er1—Er1^i^	109.54 (6)	O1^xiii^—K1—Mo1^xii^	143.10 (5)
O3^ii^—Er1—K1^iii^	138.62 (6)	O1^xiii^—K1—Mo1^xi^	81.44 (6)
O3^ix^—Er1—K1^iii^	138.62 (6)	O1^xi^—K1—Mo1	143.10 (5)
O3^ix^—Er1—O2^vii^	139.31 (8)	O1^xi^—K1—Mo1^xii^	129.86 (5)
O3^ix^—Er1—O2^iv^	76.40 (8)	O1^xiii^—K1—Mo1	129.86 (5)
O3^ii^—Er1—O2^vii^	70.94 (9)	O1^xiii^—K1—Mo1^xiii^	25.94 (5)
O3^ii^—Er1—O2^v^	76.40 (8)	O1^xi^—K1—Mo1^xiii^	81.44 (6)
O3^ix^—Er1—O2^v^	84.67 (9)	O1^xi^—K1—Mo1^xi^	25.94 (5)
O3^ix^—Er1—O2^vi^	70.94 (9)	O1^xiii^—K1—O1^xi^	59.23 (10)
O3^ii^—Er1—O2^iv^	84.67 (9)	O1^xiii^—K1—O3^xii^	132.07 (6)
O3^ii^—Er1—O2^vi^	139.31 (8)	O1^xi^—K1—O3	132.07 (6)
O3^ii^—Er1—O3^ix^	82.76 (13)	O1^xi^—K1—O3^xii^	157.57 (7)
K1—Mo1—K1^iii^	77.802 (9)	O1^xiii^—K1—O3	157.57 (7)
K1—Mo1—K1^x^	76.162 (9)	O3—K1—Mo1^xiii^	132.33 (4)
K1^iii^—Mo1—K1^x^	81.02 (2)	O3—K1—Mo1^xii^	50.39 (4)
O4—Mo1—K1^x^	36.40 (9)	O3^xii^—K1—Mo1^xi^	132.33 (4)
O4—Mo1—K1	40.51 (8)	O3^xii^—K1—Mo1^xiii^	115.60 (4)
O4—Mo1—K1^iii^	70.83 (9)	O3^xii^—K1—Mo1^xii^	28.12 (4)
O4—Mo1—O2	109.56 (12)	O3—K1—Mo1^xi^	115.60 (4)
O4—Mo1—O1	106.55 (12)	O3^xii^—K1—Mo1	50.39 (4)
O4—Mo1—O3	105.29 (12)	O3—K1—Mo1	28.12 (4)
O2—Mo1—K1^x^	86.26 (8)	O3—K1—O3^xii^	47.02 (9)
O2—Mo1—K1^iii^	155.83 (7)	Mo1—O4—K1^x^	121.94 (12)
O2—Mo1—K1	118.97 (8)	Mo1—O4—K1	114.78 (12)
O1—Mo1—K1^iii^	43.23 (8)	K1—O4—K1^x^	121.57 (10)
O1—Mo1—K1	120.81 (8)	Er1^iv^—O2—Er1^xvi^	109.14 (10)
O1—Mo1—K1^x^	95.46 (8)	Mo1—O2—Er1^xvi^	120.07 (10)
O1—Mo1—O2	118.71 (11)	Mo1—O2—Er1^iv^	127.56 (11)
O3—Mo1—K1^iii^	90.50 (8)	Er1—O1—K1^iii^	112.96 (10)
O3—Mo1—K1^x^	141.37 (9)	Mo1—O1—Er1	125.03 (13)
O3—Mo1—K1	65.21 (8)	Mo1—O1—K1^iii^	110.84 (11)
O3—Mo1—O2	111.94 (11)	Er1^ii^—O3—K1	146.22 (10)
O3—Mo1—O1	103.83 (12)	Mo1—O3—Er1^ii^	127.06 (13)
Mo1^xii^—K1—Mo1^xiii^	138.837 (12)	Mo1—O3—K1	86.67 (9)

Symmetry codes: (i) −*x*+2, −*y*+1, −*z*+2; (ii) −*x*+2, −*y*+1, −*z*+1; (iii) −*x*+3/2, −*y*+1/2, *z*+1/2; (iv) −*x*+1, −*y*+1, −*z*+1; (v) *x*+1, −*y*+1, *z*+1/2; (vi) −*x*+1, *y*, −*z*+3/2; (vii) *x*+1, *y*, *z*; (viii) −*x*+2, *y*, −*z*+3/2; (ix) *x*, −*y*+1, *z*+1/2; (x) −*x*+1/2, −*y*+1/2, *z*+1/2; (xi) −*x*+3/2, −*y*+1/2, *z*−1/2; (xii) −*x*+1, *y*, −*z*+1/2; (xiii) *x*−1/2, −*y*+1/2, −*z*+1; (xiv) *x*+1/2, −*y*+1/2, −*z*+1; (xv) −*x*+1/2, −*y*+1/2, *z*−1/2; (xvi) *x*−1, *y*, *z*.

### Potassium ytterbium bis(molybdate) (Yb_Molybdate) Crystal data

**Table:**

KYb(MoO_4_)_2_	*F*(000) = 948
*M**_r_* = 532.02	*D*_x_ = 4.866 Mg m^−^^3^
Orthorhombic, *P**b**c**n*	Mo *K*α radiation, λ = 0.71073 Å
Hall symbol: -P 2n 2ab	Cell parameters from 3007 reflections
*a* = 5.0417 (5) Å	θ = 2.2–29.2°
*b* = 18.3039 (19) Å	µ = 16.75 mm^−^^1^
*c* = 7.8693 (8) Å	*T* = 273 K
*V* = 726.20 (13) Å^3^	Plate, white
*Z* = 4	0.06 × 0.06 × 0.04 mm

### Potassium ytterbium bis(molybdate) (Yb_Molybdate) Data collection

**Table:**

Bruker APEXII CCD diffractometer	845 reflections with *I* > 2σ(*I*)
Radiation source: X-ray tube	*R*_int_ = 0.038
φ and ω scans	θ_max_ = 29.5°, θ_min_ = 2.2°
Absorption correction: multi-scan (SADABS; Krause et al., 2015)	*h* = −6→6
*T*_min_ = 0.198, *T*_max_ = 0.301	*k* = −24→25
8164 measured reflections	*l* = −10→10
978 independent reflections	

### Potassium ytterbium bis(molybdate) (Yb_Molybdate) Refinement

**Table:**

Refinement on *F*^2^	0 restraints
Least-squares matrix: full	0 constraints
*R*[*F*^2^ > 2σ(*F*^2^)] = 0.022	Primary atom site location: dual
*wR*(*F*^2^) = 0.063	*w* = 1/[σ^2^(*F*_o_^2^) + (0.0244*P*)^2^ + 3.0168*P*] where *P* = (*F*_o_^2^ + 2*F*_c_^2^)/3
*S* = 1.11	(Δ/σ)_max_ = 0.001
978 reflections	Δρ_max_ = 1.55 e Å^−^^3^
56 parameters	Δρ_min_ = −1.14 e Å^−^^3^

### Potassium ytterbium bis(molybdate) (Yb_Molybdate) Special details

**Table:**

Geometry. All esds (except the esd in the dihedral angle between two l.s. planes) are estimated using the full covariance matrix. The cell esds are taken into account individually in the estimation of esds in distances, angles and torsion angles; correlations between esds in cell parameters are only used when they are defined by crystal symmetry. An approximate (isotropic) treatment of cell esds is used for estimating esds involving l.s. planes.
Refinement. Reflections were merged by SHELXL according to the crystal class for the calculation of statistics and refinement. _reflns_Friedel_fraction is defined as the number of unique Friedel pairs measured divided by the number that would be possible theoretically, ignoring centric projections and systematic absences.

### Potassium ytterbium bis(molybdate) (Yb_Molybdate) Fractional atomic coordinates and isotropic or equivalent isotropic displacement parameters (Å^2^)

**Table:**

	*x*	*y*	*z*	*U*_iso_*/*U*_eq_	
Yb1	0.000000	0.49506 (2)	0.750000	0.01036 (11)	
Mo1	0.47826 (7)	0.60027 (2)	0.51395 (5)	0.01066 (12)	
K1	0.500000	0.77252 (9)	0.250000	0.0230 (3)	
O4	0.6053 (7)	0.68778 (18)	0.5206 (4)	0.0188 (7)	
O2	0.7521 (6)	0.53396 (17)	0.4939 (3)	0.0128 (6)	
O1	0.2572 (6)	0.59262 (17)	0.6912 (4)	0.0149 (6)	
O3	0.2661 (6)	0.59860 (17)	0.3360 (4)	0.0152 (6)	

### Potassium ytterbium bis(molybdate) (Yb_Molybdate) Atomic displacement parameters (Å^2^)

**Table:**

	*U*^11^	*U*^22^	*U*^33^	*U*^12^	*U*^13^	*U*^23^
Yb1	0.00810 (17)	0.01404 (17)	0.00894 (16)	0.000	0.00084 (9)	0.000
Mo1	0.0085 (2)	0.0117 (2)	0.0118 (2)	0.00068 (12)	0.00134 (12)	0.00054 (13)
K1	0.0306 (9)	0.0195 (7)	0.0189 (7)	0.000	0.0019 (6)	0.000
O4	0.0212 (18)	0.0179 (17)	0.0173 (16)	−0.0025 (14)	−0.0044 (13)	0.0007 (13)
O2	0.0112 (15)	0.0184 (17)	0.0086 (14)	0.0033 (12)	0.0023 (12)	−0.0010 (11)
O1	0.0131 (14)	0.0175 (15)	0.0143 (15)	−0.0011 (12)	0.0039 (12)	−0.0038 (13)
O3	0.0142 (15)	0.0177 (16)	0.0137 (15)	−0.0024 (13)	0.0006 (12)	0.0003 (12)

### Potassium ytterbium bis(molybdate) (Yb_Molybdate) Geometric parameters (Å, º)

**Table:**

Yb1—Mo1^i^	3.6018 (5)	Mo1—O2	1.845 (3)
Yb1—Mo1^ii^	3.6294 (5)	Mo1—O1	1.791 (3)
Yb1—Mo1^iii^	3.6294 (5)	Mo1—O3	1.762 (3)
Yb1—Mo1	3.6018 (5)	K1—Mo1^xi^	3.9738 (11)
Yb1—O2^iv^	2.351 (3)	K1—Mo1^xii^	3.7772 (14)
Yb1—O2^v^	2.476 (3)	K1—Mo1^xiii^	3.8322 (11)
Yb1—O2^vi^	2.351 (3)	K1—Mo1^xiv^	3.8322 (11)
Yb1—O2^vii^	2.476 (3)	K1—Mo1^xv^	3.9738 (11)
Yb1—O1	2.255 (3)	K1—O4^xii^	2.687 (3)
Yb1—O1^i^	2.255 (3)	K1—O4	2.687 (3)
Yb1—O3^ii^	2.280 (3)	K1—O4^xi^	2.784 (3)
Yb1—O3^iii^	2.280 (3)	K1—O4^xv^	2.784 (3)
Mo1—Yb1^iii^	3.6294 (5)	K1—O1^xiii^	2.826 (3)
Mo1—Yb1^iv^	3.7786 (5)	K1—O1^xiv^	2.826 (3)
Mo1—Yb1^viii^	3.7522 (5)	O4—K1^x^	2.784 (3)
Mo1—K1^ix^	3.8322 (11)	O2—Yb1^iv^	2.351 (3)
Mo1—K1^x^	3.9738 (11)	O2—Yb1^viii^	2.476 (3)
Mo1—K1	3.7772 (14)	O1—K1^ix^	2.826 (3)
Mo1—O4	1.726 (3)	O3—Yb1^iii^	2.280 (3)
			
Mo1—Yb1—Mo1^i^	115.356 (17)	O2—Mo1—Yb1	101.15 (10)
Mo1^i^—Yb1—Mo1^iii^	96.169 (13)	O2—Mo1—Yb1^iii^	97.58 (10)
Mo1—Yb1—Mo1^iii^	113.994 (10)	O2—Mo1—K1^ix^	155.78 (8)
Mo1—Yb1—Mo1^ii^	96.168 (13)	O2—Mo1—K1^x^	85.99 (10)
Mo1^iii^—Yb1—Mo1^ii^	122.531 (16)	O2—Mo1—K1	118.71 (10)
Mo1^i^—Yb1—Mo1^ii^	113.994 (10)	O1—Mo1—Yb1^iii^	89.70 (10)
O2^v^—Yb1—Mo1^i^	85.88 (7)	O1—Mo1—Yb1^viii^	90.61 (10)
O2^iv^—Yb1—Mo1^ii^	90.31 (8)	O1—Mo1—Yb1^iv^	145.62 (10)
O2^vii^—Yb1—Mo1^i^	76.37 (8)	O1—Mo1—Yb1	30.68 (10)
O2^iv^—Yb1—Mo1	49.00 (8)	O1—Mo1—K1	120.77 (10)
O2^vii^—Yb1—Mo1^ii^	48.48 (8)	O1—Mo1—K1^x^	95.37 (10)
O2^iv^—Yb1—Mo1^iii^	77.14 (8)	O1—Mo1—K1^ix^	43.81 (10)
O2^vi^—Yb1—Mo1^i^	49.00 (8)	O1—Mo1—O2	118.75 (14)
O2^vi^—Yb1—Mo1	153.85 (8)	O3—Mo1—Yb1	89.67 (10)
O2^vi^—Yb1—Mo1^ii^	77.14 (8)	O3—Mo1—Yb1^viii^	144.09 (11)
O2^vii^—Yb1—Mo1	85.88 (7)	O3—Mo1—Yb1^iii^	29.97 (10)
O2^v^—Yb1—Mo1^ii^	159.95 (7)	O3—Mo1—Yb1^iv^	88.72 (10)
O2^v^—Yb1—Mo1^iii^	48.48 (8)	O3—Mo1—K1	66.11 (10)
O2^iv^—Yb1—Mo1^i^	153.85 (8)	O3—Mo1—K1^x^	141.56 (11)
O2^vi^—Yb1—Mo1^iii^	90.31 (8)	O3—Mo1—K1^ix^	90.79 (10)
O2^vii^—Yb1—Mo1^iii^	159.95 (7)	O3—Mo1—O2	112.01 (14)
O2^v^—Yb1—Mo1	76.37 (8)	O3—Mo1—O1	103.88 (15)
O2^iv^—Yb1—O2^v^	70.66 (12)	Mo1^xiv^—K1—Mo1^xv^	80.44 (3)
O2^iv^—Yb1—O2^vii^	117.45 (14)	Mo1^xii^—K1—Mo1	66.83 (3)
O2^vi^—Yb1—O2^vii^	70.66 (12)	Mo1—K1—Mo1^xv^	139.938 (17)
O2^vi^—Yb1—O2^v^	117.45 (14)	Mo1^xiii^—K1—Mo1^xiv^	105.17 (4)
O2^vii^—Yb1—O2^v^	146.58 (15)	Mo1—K1—Mo1^xi^	102.288 (13)
O2^iv^—Yb1—O2^vi^	153.89 (16)	Mo1—K1—Mo1^xiii^	105.003 (13)
O1—Yb1—Mo1	23.91 (8)	Mo1^xii^—K1—Mo1^xv^	102.287 (13)
O1^i^—Yb1—Mo1^iii^	96.67 (8)	Mo1^xii^—K1—Mo1^xiii^	139.065 (16)
O1—Yb1—Mo1^ii^	96.67 (8)	Mo1^xiii^—K1—Mo1^xv^	56.91 (2)
O1^i^—Yb1—Mo1	93.86 (8)	Mo1^xiv^—K1—Mo1^xi^	56.91 (2)
O1^i^—Yb1—Mo1^ii^	130.19 (8)	Mo1^xii^—K1—Mo1^xi^	139.938 (17)
O1—Yb1—Mo1^i^	93.86 (8)	Mo1^xii^—K1—Mo1^xiv^	105.003 (13)
O1^i^—Yb1—Mo1^i^	23.91 (8)	Mo1^xiii^—K1—Mo1^xi^	80.44 (3)
O1—Yb1—Mo1^iii^	130.19 (8)	Mo1—K1—Mo1^xiv^	139.065 (16)
O1^i^—Yb1—O2^v^	69.45 (10)	Mo1^xv^—K1—Mo1^xi^	108.26 (4)
O1—Yb1—O2^vii^	69.45 (10)	O4—K1—Mo1^xiv^	149.21 (8)
O1^i^—Yb1—O2^iv^	130.70 (11)	O4^xi^—K1—Mo1^xi^	21.66 (7)
O1—Yb1—O2^iv^	72.89 (11)	O4—K1—Mo1^xiii^	80.93 (7)
O1^i^—Yb1—O2^vii^	84.00 (11)	O4^xi^—K1—Mo1^xiv^	72.90 (7)
O1—Yb1—O2^v^	84.00 (11)	O4^xi^—K1—Mo1^xii^	126.53 (8)
O1—Yb1—O2^vi^	130.70 (11)	O4^xii^—K1—Mo1	87.03 (8)
O1^i^—Yb1—O2^vi^	72.89 (11)	O4^xii^—K1—Mo1^xi^	125.30 (8)
O1—Yb1—O1^i^	75.25 (16)	O4—K1—Mo1	24.24 (7)
O1^i^—Yb1—O3^iii^	108.54 (12)	O4^xv^—K1—Mo1^xiii^	72.90 (7)
O1—Yb1—O3^iii^	150.82 (12)	O4^xi^—K1—Mo1	80.82 (7)
O1^i^—Yb1—O3^ii^	150.82 (12)	O4^xii^—K1—Mo1^xiii^	149.21 (8)
O1—Yb1—O3^ii^	108.54 (12)	O4^xv^—K1—Mo1	126.53 (8)
O3^iii^—Yb1—Mo1	130.01 (7)	O4^xi^—K1—Mo1^xiii^	88.68 (8)
O3^ii^—Yb1—Mo1^iii^	101.50 (8)	O4^xii^—K1—Mo1^xv^	95.65 (7)
O3^ii^—Yb1—Mo1^i^	130.01 (7)	O4—K1—Mo1^xi^	95.65 (7)
O3^ii^—Yb1—Mo1	99.27 (8)	O4—K1—Mo1^xv^	125.30 (8)
O3^iii^—Yb1—Mo1^i^	99.27 (8)	O4^xv^—K1—Mo1^xi^	128.58 (9)
O3^iii^—Yb1—Mo1^iii^	22.72 (8)	O4^xi^—K1—Mo1^xv^	128.58 (9)
O3^iii^—Yb1—Mo1^ii^	101.50 (8)	O4—K1—Mo1^xii^	87.03 (8)
O3^ii^—Yb1—Mo1^ii^	22.72 (8)	O4^xv^—K1—Mo1^xv^	21.66 (7)
O3^iii^—Yb1—O2^iv^	84.30 (11)	O4^xv^—K1—Mo1^xii^	80.82 (7)
O3^ii^—Yb1—O2^v^	139.02 (10)	O4^xii^—K1—Mo1^xii^	24.25 (7)
O3^iii^—Yb1—O2^v^	71.18 (11)	O4^xv^—K1—Mo1^xiv^	88.68 (8)
O3^ii^—Yb1—O2^iv^	76.09 (10)	O4^xii^—K1—Mo1^xiv^	80.93 (7)
O3^ii^—Yb1—O2^vi^	84.30 (11)	O4^xii^—K1—O4^xi^	121.56 (5)
O3^iii^—Yb1—O2^vi^	76.09 (10)	O4^xii^—K1—O4	109.49 (15)
O3^iii^—Yb1—O2^vii^	139.02 (10)	O4—K1—O4^xv^	121.56 (5)
O3^ii^—Yb1—O2^vii^	71.18 (11)	O4—K1—O4^xi^	77.17 (8)
O3^iii^—Yb1—O3^ii^	82.48 (16)	O4^xii^—K1—O4^xv^	77.17 (8)
Yb1^viii^—Mo1—Yb1^iv^	63.070 (9)	O4^xi^—K1—O4^xv^	149.74 (15)
Yb1^iii^—Mo1—Yb1^viii^	120.155 (13)	O4^xv^—K1—O1^xiv^	89.65 (10)
Yb1^iii^—Mo1—Yb1^iv^	85.752 (11)	O4^xv^—K1—O1^xiii^	63.27 (9)
Yb1—Mo1—Yb1^iii^	66.007 (10)	O4^xii^—K1—O1^xiv^	106.49 (10)
Yb1—Mo1—Yb1^viii^	86.536 (12)	O4^xi^—K1—O1^xiii^	89.65 (10)
Yb1—Mo1—Yb1^iv^	120.176 (13)	O4^xi^—K1—O1^xiv^	63.27 (9)
Yb1—Mo1—K1	138.532 (12)	O4^xii^—K1—O1^xiii^	136.42 (10)
Yb1^iii^—Mo1—K1^x^	171.48 (2)	O4—K1—O1^xiii^	106.49 (10)
Yb1^iv^—Mo1—K1^ix^	170.07 (2)	O4—K1—O1^xiv^	136.42 (10)
Yb1^iii^—Mo1—K1	96.075 (15)	O1^xiv^—K1—Mo1^xiv^	26.02 (6)
Yb1^viii^—Mo1—K1^ix^	120.864 (13)	O1^xiv^—K1—Mo1^xiii^	80.64 (7)
Yb1^viii^—Mo1—K1	132.869 (12)	O1^xiii^—K1—Mo1^xi^	73.47 (7)
Yb1—Mo1—K1^x^	121.021 (14)	O1^xiii^—K1—Mo1^xiv^	80.64 (7)
Yb1^iv^—Mo1—K1^x^	93.824 (14)	O1^xiii^—K1—Mo1	130.72 (6)
Yb1^viii^—Mo1—K1^x^	66.75 (2)	O1^xiii^—K1—Mo1^xii^	143.68 (7)
Yb1—Mo1—K1^ix^	69.74 (2)	O1^xiv^—K1—Mo1^xi^	42.36 (7)
Yb1^iii^—Mo1—K1^ix^	98.712 (15)	O1^xiv^—K1—Mo1^xii^	130.72 (6)
K1—Mo1—Yb1^iv^	93.608 (16)	O1^xiv^—K1—Mo1^xv^	73.47 (7)
K1—Mo1—K1^ix^	77.153 (12)	O1^xiii^—K1—Mo1^xv^	42.36 (7)
K1—Mo1—K1^x^	75.448 (12)	O1^xiv^—K1—Mo1	143.68 (7)
K1^ix^—Mo1—K1^x^	80.44 (3)	O1^xiii^—K1—Mo1^xiii^	26.02 (6)
O4—Mo1—Yb1	136.81 (11)	O1^xiii^—K1—O1^xiv^	58.30 (13)
O4—Mo1—Yb1^viii^	101.62 (11)	Mo1—O4—K1	116.01 (15)
O4—Mo1—Yb1^iv^	100.77 (12)	Mo1—O4—K1^x^	121.81 (16)
O4—Mo1—Yb1^iii^	135.23 (11)	K1—O4—K1^x^	120.30 (12)
O4—Mo1—K1^x^	36.53 (11)	Yb1^iv^—O2—Yb1^viii^	109.33 (12)
O4—Mo1—K1^ix^	69.81 (12)	Mo1—O2—Yb1^iv^	128.04 (14)
O4—Mo1—K1	39.74 (10)	Mo1—O2—Yb1^viii^	119.82 (13)
O4—Mo1—O2	109.61 (16)	Yb1—O1—K1^ix^	113.22 (12)
O4—Mo1—O1	106.25 (15)	Mo1—O1—Yb1	125.42 (16)
O4—Mo1—O3	105.38 (15)	Mo1—O1—K1^ix^	110.17 (14)
O2—Mo1—Yb1^iv^	29.34 (8)	Mo1—O3—Yb1^iii^	127.31 (16)
O2—Mo1—Yb1^viii^	34.93 (8)		

Symmetry codes: (i) −*x*, *y*, −*z*+3/2; (ii) *x*, −*y*+1, *z*+1/2; (iii) −*x*, −*y*+1, −*z*+1; (iv) −*x*+1, −*y*+1, −*z*+1; (v) *x*−1, *y*, *z*; (vi) *x*−1, −*y*+1, *z*+1/2; (vii) −*x*+1, *y*, −*z*+3/2; (viii) *x*+1, *y*, *z*; (ix) −*x*+1/2, −*y*+3/2, *z*+1/2; (x) −*x*+3/2, −*y*+3/2, *z*+1/2; (xi) *x*−1/2, −*y*+3/2, −*z*+1; (xii) −*x*+1, *y*, −*z*+1/2; (xiii) *x*+1/2, −*y*+3/2, −*z*+1; (xiv) −*x*+1/2, −*y*+3/2, *z*−1/2; (xv) −*x*+3/2, −*y*+3/2, *z*−1/2.

### Potassium lutetium bis(molybdate) (Lu_Molybdate) Crystal data

**Table:**

KLu(MoO_4_)_2_	*F*(000) = 952
*M**_r_* = 533.95	*D*_x_ = 4.942 Mg m^−^^3^
Orthorhombic, *P**b**c**n*	Mo *K*α radiation, λ = 0.71073 Å
Hall symbol: -P 2n 2ab	Cell parameters from 6634 reflections
*a* = 5.0292 (2) Å	θ = 3.4–30.5°
*b* = 18.2519 (10) Å	µ = 17.68 mm^−^^1^
*c* = 7.8174 (4) Å	*T* = 100 K
*V* = 717.58 (6) Å^3^	Plate, white
*Z* = 4	0.44 × 0.14 × 0.04 mm

### Potassium lutetium bis(molybdate) (Lu_Molybdate) Data collection

**Table:**

Rigaku XtaLAB Synergy-S, HyPix diffractometer	1105 independent reflections
Radiation source: micro-focus sealed X-ray tube	1004 reflections with *I* > 2σ(*I*)
Mirror monochromator	*R*_int_ = 0.087
Detector resolution: 10.0000 pixels mm^-1^	θ_max_ = 30.5°, θ_min_ = 2.2°
ω scans	*h* = −7→7
Absorption correction: multi-scan (CrysAlisPro; Rigaku OD, 2019)	*k* = −26→26
*T*_min_ = 0.240, *T*_max_ = 1.000	*l* = −11→11
19857 measured reflections	

### Potassium lutetium bis(molybdate) (Lu_Molybdate) Refinement

**Table:**

Refinement on *F*^2^	0 restraints
Least-squares matrix: full	0 constraints
*R*[*F*^2^ > 2σ(*F*^2^)] = 0.027	Primary atom site location: dual
*wR*(*F*^2^) = 0.077	*w* = 1/[σ^2^(*F*_o_^2^) + (0.0305*P*)^2^ + 4.6788*P*] where *P* = (*F*_o_^2^ + 2*F*_c_^2^)/3
*S* = 1.17	(Δ/σ)_max_ = 0.001
1105 reflections	Δρ_max_ = 2.05 e Å^−^^3^
57 parameters	Δρ_min_ = −3.03 e Å^−^^3^

### Potassium lutetium bis(molybdate) (Lu_Molybdate) Special details

**Table:**

Geometry. All esds (except the esd in the dihedral angle between two l.s. planes) are estimated using the full covariance matrix. The cell esds are taken into account individually in the estimation of esds in distances, angles and torsion angles; correlations between esds in cell parameters are only used when they are defined by crystal symmetry. An approximate (isotropic) treatment of cell esds is used for estimating esds involving l.s. planes.
Refinement. Reflections were merged by SHELXL according to the crystal class for the calculation of statistics and refinement. _reflns_Friedel_fraction is defined as the number of unique Friedel pairs measured divided by the number that would be possible theoretically, ignoring centric projections and systematic absences.

### Potassium lutetium bis(molybdate) (Lu_Molybdate) Fractional atomic coordinates and isotropic or equivalent isotropic displacement parameters (Å^2^)

**Table:**

	*x*	*y*	*z*	*U*_iso_*/*U*_eq_	
Lu1	1.000000	0.50463 (2)	0.750000	0.00674 (13)	
Mo1	0.52103 (8)	0.39975 (3)	0.51329 (5)	0.00699 (13)	
K1	0.500000	0.22690 (9)	0.250000	0.0116 (3)	
O4	0.3935 (7)	0.31279 (19)	0.5200 (4)	0.0119 (7)	
O2	0.2476 (7)	0.4667 (2)	0.4950 (4)	0.0098 (7)	
O1	0.7429 (6)	0.40718 (19)	0.6920 (4)	0.0101 (6)	
O3	0.7347 (6)	0.40152 (19)	0.3343 (4)	0.0098 (6)	

### Potassium lutetium bis(molybdate) (Lu_Molybdate) Atomic displacement parameters (Å^2^)

**Table:**

	*U*^11^	*U*^22^	*U*^33^	*U*^12^	*U*^13^	*U*^23^
Lu1	0.00628 (18)	0.0100 (2)	0.00389 (17)	0.000	0.00006 (8)	0.000
Mo1	0.0067 (2)	0.0091 (2)	0.0051 (2)	0.00028 (12)	−0.00024 (11)	−0.00031 (13)
K1	0.0142 (7)	0.0132 (8)	0.0073 (6)	0.000	−0.0006 (4)	0.000
O4	0.0128 (17)	0.0141 (17)	0.0087 (14)	−0.0011 (13)	0.0006 (12)	−0.0018 (12)
O2	0.0079 (15)	0.0168 (18)	0.0046 (14)	0.0006 (13)	−0.0051 (11)	0.0004 (11)
O1	0.0100 (14)	0.0130 (16)	0.0074 (14)	−0.0011 (13)	−0.0028 (12)	0.0009 (12)
O3	0.0118 (15)	0.0126 (16)	0.0051 (13)	0.0008 (13)	0.0002 (11)	−0.0001 (11)

### Potassium lutetium bis(molybdate) (Lu_Molybdate) Geometric parameters (Å, º)

**Table:**

Lu1—Lu1^i^	3.9123 (2)	Mo1—O3	1.765 (3)
Lu1—Lu1^ii^	3.9123 (2)	K1—Mo1^xi^	3.9540 (11)
Lu1—K1^iii^	4.2257 (17)	K1—Mo1^xii^	3.7684 (14)
Lu1—O2^iv^	2.344 (3)	K1—Mo1^xiii^	3.8171 (11)
Lu1—O2^v^	2.344 (3)	K1—Mo1^xiv^	3.8171 (11)
Lu1—O2^vi^	2.450 (3)	K1—Mo1^xv^	3.9540 (11)
Lu1—O2^vii^	2.450 (3)	K1—O4	2.683 (3)
Lu1—O1	2.245 (3)	K1—O4^xii^	2.683 (3)
Lu1—O1^viii^	2.245 (3)	K1—O4^xv^	2.770 (3)
Lu1—O3^ix^	2.269 (3)	K1—O4^xi^	2.770 (3)
Lu1—O3^ii^	2.269 (3)	K1—O1^xiii^	2.805 (4)
Mo1—K1	3.7684 (14)	K1—O1^xiv^	2.805 (4)
Mo1—K1^x^	3.9540 (11)	O4—K1^x^	2.770 (3)
Mo1—K1^iii^	3.8171 (11)	O2—Lu1^iv^	2.344 (3)
Mo1—O4	1.713 (4)	O2—Lu1^xvi^	2.450 (3)
Mo1—O2	1.845 (4)	O1—K1^iii^	2.805 (4)
Mo1—O1	1.793 (3)	O3—Lu1^ii^	2.269 (3)
			
Lu1^ii^—Lu1—Lu1^i^	175.051 (17)	Mo1^xiv^—K1—Mo1^xv^	80.64 (3)
Lu1^ii^—Lu1—K1^iii^	87.526 (9)	Mo1—K1—Mo1^xv^	139.788 (18)
Lu1^i^—Lu1—K1^iii^	87.526 (9)	Mo1^xiii^—K1—Mo1^xiv^	105.46 (4)
O2^vii^—Lu1—Lu1^ii^	34.41 (8)	Mo1^xii^—K1—Mo1^xi^	139.788 (18)
O2^vii^—Lu1—Lu1^i^	143.18 (8)	Mo1^xii^—K1—Mo1	66.31 (3)
O2^iv^—Lu1—Lu1^ii^	36.22 (8)	Mo1^xiii^—K1—Mo1^xi^	80.64 (3)
O2^v^—Lu1—Lu1^ii^	145.70 (8)	Mo1^xii^—K1—Mo1^xv^	102.429 (13)
O2^vi^—Lu1—Lu1^i^	34.41 (8)	Mo1—K1—Mo1^xiii^	105.066 (13)
O2^v^—Lu1—Lu1^i^	36.22 (8)	Mo1^xiii^—K1—Mo1^xv^	56.95 (2)
O2^iv^—Lu1—Lu1^i^	145.70 (8)	Mo1^xii^—K1—Mo1^xiv^	105.066 (14)
O2^vi^—Lu1—Lu1^ii^	143.18 (8)	Mo1—K1—Mo1^xiv^	138.944 (16)
O2^vii^—Lu1—K1^iii^	73.58 (8)	Mo1—K1—Mo1^xi^	102.429 (13)
O2^iv^—Lu1—K1^iii^	102.92 (9)	Mo1^xiv^—K1—Mo1^xi^	56.95 (2)
O2^v^—Lu1—K1^iii^	102.92 (9)	Mo1^xii^—K1—Mo1^xiii^	138.944 (16)
O2^vi^—Lu1—K1^iii^	73.58 (8)	Mo1^xv^—K1—Mo1^xi^	108.45 (4)
O2^iv^—Lu1—O2^vi^	117.26 (15)	O4^xii^—K1—Mo1^xiv^	81.17 (8)
O2^v^—Lu1—O2^vii^	117.26 (15)	O4^xv^—K1—Mo1^xi^	128.63 (9)
O2^v^—Lu1—O2^iv^	154.16 (18)	O4^xv^—K1—Mo1^xii^	81.05 (7)
O2^v^—Lu1—O2^vi^	70.63 (14)	O4^xv^—K1—Mo1^xiv^	88.73 (8)
O2^vii^—Lu1—O2^vi^	147.15 (17)	O4—K1—Mo1	24.06 (8)
O2^iv^—Lu1—O2^vii^	70.63 (14)	O4^xi^—K1—Mo1^xiv^	72.87 (7)
O1—Lu1—Lu1^i^	99.65 (8)	O4—K1—Mo1^xi^	96.06 (8)
O1—Lu1—Lu1^ii^	76.35 (8)	O4^xii^—K1—Mo1	86.27 (8)
O1^viii^—Lu1—Lu1^i^	76.35 (8)	O4^xi^—K1—Mo1^xiii^	88.73 (8)
O1^viii^—Lu1—Lu1^ii^	99.65 (8)	O4^xv^—K1—Mo1	126.40 (8)
O1^viii^—Lu1—K1^iii^	37.61 (8)	O4^xii^—K1—Mo1^xii^	24.06 (8)
O1—Lu1—K1^iii^	37.61 (8)	O4^xi^—K1—Mo1	81.05 (7)
O1^viii^—Lu1—O2^iv^	130.40 (12)	O4^xv^—K1—Mo1^xiii^	72.87 (7)
O1—Lu1—O2^vi^	69.37 (11)	O4—K1—Mo1^xv^	125.27 (8)
O1^viii^—Lu1—O2^vii^	69.37 (11)	O4^xii^—K1—Mo1^xi^	125.27 (8)
O1—Lu1—O2^iv^	72.91 (12)	O4^xii^—K1—Mo1^xv^	96.06 (8)
O1^viii^—Lu1—O2^v^	72.91 (12)	O4^xi^—K1—Mo1^xi^	21.55 (7)
O1—Lu1—O2^vii^	84.51 (12)	O4^xv^—K1—Mo1^xv^	21.55 (7)
O1—Lu1—O2^v^	130.40 (12)	O4^xi^—K1—Mo1^xii^	126.40 (8)
O1^viii^—Lu1—O2^vi^	84.51 (12)	O4^xi^—K1—Mo1^xv^	128.63 (9)
O1^viii^—Lu1—O1	75.22 (17)	O4—K1—Mo1^xiv^	149.44 (8)
O1—Lu1—O3^ix^	108.54 (13)	O4—K1—Mo1^xii^	86.27 (8)
O1^viii^—Lu1—O3^ix^	151.41 (12)	O4^xii^—K1—Mo1^xiii^	149.44 (8)
O1^viii^—Lu1—O3^ii^	108.54 (13)	O4—K1—Mo1^xiii^	81.17 (8)
O1—Lu1—O3^ii^	151.41 (12)	O4—K1—O4^xv^	121.38 (5)
O3^ii^—Lu1—Lu1^ii^	75.08 (8)	O4—K1—O4^xii^	108.51 (16)
O3^ix^—Lu1—Lu1^ii^	108.82 (8)	O4^xii^—K1—O4^xi^	121.38 (5)
O3^ii^—Lu1—Lu1^i^	108.82 (8)	O4^xii^—K1—O4^xv^	77.57 (9)
O3^ix^—Lu1—Lu1^i^	75.08 (8)	O4—K1—O4^xi^	77.57 (9)
O3^ii^—Lu1—K1^iii^	139.02 (8)	O4^xv^—K1—O4^xi^	149.68 (16)
O3^ix^—Lu1—K1^iii^	139.02 (8)	O4^xi^—K1—O1^xiv^	63.09 (10)
O3^ii^—Lu1—O2^vii^	71.24 (12)	O4^xi^—K1—O1^xiii^	89.78 (11)
O3^ii^—Lu1—O2^iv^	84.63 (12)	O4—K1—O1^xiv^	136.80 (10)
O3^ix^—Lu1—O2^v^	84.63 (12)	O4^xv^—K1—O1^xiii^	63.09 (10)
O3^ii^—Lu1—O2^v^	75.88 (11)	O4^xv^—K1—O1^xiv^	89.78 (11)
O3^ix^—Lu1—O2^vii^	138.46 (11)	O4—K1—O1^xiii^	106.89 (10)
O3^ii^—Lu1—O2^vi^	138.46 (11)	O4^xii^—K1—O1^xiii^	136.80 (10)
O3^ix^—Lu1—O2^vi^	71.24 (12)	O4^xii^—K1—O1^xiv^	106.89 (10)
O3^ix^—Lu1—O2^iv^	75.88 (11)	O1^xiv^—K1—Mo1^xiv^	26.14 (7)
O3^ii^—Lu1—O3^ix^	81.96 (17)	O1^xiv^—K1—Mo1^xiii^	80.84 (7)
K1—Mo1—K1^x^	75.379 (13)	O1^xiii^—K1—Mo1^xi^	73.72 (7)
K1—Mo1—K1^iii^	77.027 (12)	O1^xiii^—K1—Mo1^xiv^	80.84 (7)
K1^iii^—Mo1—K1^x^	80.64 (3)	O1^xiii^—K1—Mo1	130.93 (6)
O4—Mo1—K1	39.69 (11)	O1^xiii^—K1—Mo1^xii^	143.70 (7)
O4—Mo1—K1^x^	36.45 (11)	O1^xiv^—K1—Mo1^xi^	42.27 (7)
O4—Mo1—K1^iii^	70.14 (12)	O1^xiv^—K1—Mo1^xii^	130.93 (6)
O4—Mo1—O2	109.68 (17)	O1^xiv^—K1—Mo1^xv^	73.72 (7)
O4—Mo1—O1	106.21 (16)	O1^xiii^—K1—Mo1^xv^	42.27 (7)
O4—Mo1—O3	105.63 (16)	O1^xiv^—K1—Mo1	143.70 (7)
O2—Mo1—K1^iii^	155.36 (9)	O1^xiii^—K1—Mo1^xiii^	26.14 (7)
O2—Mo1—K1	119.41 (10)	O1^xiii^—K1—O1^xiv^	58.48 (14)
O2—Mo1—K1^x^	85.95 (11)	Mo1—O4—K1	116.25 (16)
O1—Mo1—K1^iii^	43.56 (11)	Mo1—O4—K1^x^	122.00 (16)
O1—Mo1—K1^x^	95.28 (11)	K1—O4—K1^x^	120.03 (13)
O1—Mo1—K1	120.42 (11)	Lu1^iv^—O2—Lu1^xvi^	109.37 (14)
O1—Mo1—O2	118.31 (14)	Mo1—O2—Lu1^iv^	127.40 (15)
O3—Mo1—K1^x^	141.76 (12)	Mo1—O2—Lu1^xvi^	120.19 (14)
O3—Mo1—K1^iii^	90.65 (11)	Lu1—O1—K1^iii^	113.15 (12)
O3—Mo1—K1	66.38 (11)	Mo1—O1—Lu1	125.15 (17)
O3—Mo1—O2	112.35 (14)	Mo1—O1—K1^iii^	110.29 (15)
O3—Mo1—O1	103.74 (16)	Mo1—O3—Lu1^ii^	127.02 (17)

Symmetry codes: (i) −*x*+2, −*y*+1, −*z*+2; (ii) −*x*+2, −*y*+1, −*z*+1; (iii) −*x*+3/2, −*y*+1/2, *z*+1/2; (iv) −*x*+1, −*y*+1, −*z*+1; (v) *x*+1, −*y*+1, *z*+1/2; (vi) −*x*+1, *y*, −*z*+3/2; (vii) *x*+1, *y*, *z*; (viii) −*x*+2, *y*, −*z*+3/2; (ix) *x*, −*y*+1, *z*+1/2; (x) −*x*+1/2, −*y*+1/2, *z*+1/2; (xi) *x*+1/2, −*y*+1/2, −*z*+1; (xii) −*x*+1, *y*, −*z*+1/2; (xiii) *x*−1/2, −*y*+1/2, −*z*+1; (xiv) −*x*+3/2, −*y*+1/2, *z*−1/2; (xv) −*x*+1/2, −*y*+1/2, *z*−1/2; (xvi) *x*−1, *y*, *z*.
