# Supplementary material for: Crystal structures and comparisons of potassium rare-earth molybdates KRE(MoO4)2 (RE = Tb, Dy, Ho, Er, Yb, and Lu)
Source: Acta Crystallogr E Crystallogr Commun. 2020 Nov 27;76(Pt 12):1871–5. doi: 10.1107/S205698902001542X (PMC7784660; doi:10.1107/S205698902001542X)
Supplement: Supplementary file 8 [file e-76-01871-sup8.pdf]

Bond-valence tables of K*RE*(MoO_4)$_2$ compounds. Bond-valence units are in valence unit and length are in Å. R_0 and B values are from Brown and Altermatt, (1985), Acta Cryst. B41, 244-247 and Brese and O'Keeffe, (1991), Acta Cryst. B47, 192-197.

KTb(MoO_4)$_2$

Bond Valence Calculations					
	Tb_1	Mo_1	K_1	Sum	V - Sum
O_1	0.949	1.361	0.314	1.99	-0.01
O_2	1.290	1.176		1.82	-0.18
O_3	0.872	1.460	0.078	1.90	-0.10
O_4		1.662	0.806	2.06	0.06
Sum	3.11	5.66	1.20		

Bond Lengths			
	Tb_1	Mo_1	K_1
O_1	2.308	1.793	2.817
O_2	2.399, 2.511	1.847	
O_3	2.339	1.767	3.330
O_4		1.719	2.684, 2.771

	Tb-O	Mo-O	K-O
R_0	2.032	1.907	2.132
B	0.37	0.37	0.37

$KDy(MoO_4)_2$

Bond Valence Calculations					
	Dy_1	Mo_1	K_1	Sum	V - Sum
O_1	0.911	1.354	0.318	1.97	-0.03
O_2	1.227	1.182		1.80	-0.20
O_3	0.833	1.464	0.074	1.92	-0.08
O_4		1.658	0.816	2.07	0.07
Sum	2.97	5.66	1.21		

Bond Lengths			
	Dy_1	Mo_1	K_1
O_1	2.292	1.795	2.812
O_2	2.384, 2.502	1.845	
O_3	2.325	1.766	3.352
O_4		1.720	2.770, 2.676

	Dy-O	Mo-O	K-O
R_0	2.001	1.907	2.132
B	0.37	0.37	0.37

$KHo(MoO_4)_2$

Bond Valence Calculations					
	Ho_1	Mo_1	K_1	Sum	V - Sum
O_1	0.999	1.346	0.312	2.00	0.00
O_2	1.335	1.157		1.82	-0.18
O_3	0.916	1.480	0.069	1.97	-0.03
O_4		1.640	0.807	2.07	0.07
Sum	3.25	5.62	1.19		

Bond Lengths			
	Ho_1	Mo_1	K_1
O_1	2.282	1.797	2.819
O_2	2.372, 2.501	1.853	
O_3	2.314	1.762	3.380
O_4		1.724	2.775, 2.680

	Ho-O	Mo-O	K-O
R_0	2.025	1.907	2.132
B	0.37	0.37	0.37

$KEr(MoO_4)_2$

Bond Valence Calculations					
	Er_1	Mo_1	K_1	Sum	V - Sum
O_1	0.906	1.368	0.324	1.98	-0.02
O_2	1.244	1.176		1.80	-0.20
O_3	0.854	1.460	0.064	1.92	-0.08
O_4		1.644	0.815	2.05	0.05
Sum	3.00	5.65	1.20		

Bond Lengths			
	Er_1	Mo_1	K_1
O_1	2.281	1.791	2.805
O_2	2.370, 2.478	1.847	
O_3	2.303	1.767	3.403
O_4		1.723	2.770, 2.677

	Er-O	Mo-O	K-O
R_0	1.988	1.907	2.132
B	0.37	0.37	0.37

$KYb(MoO_4)_2$

Bond Valence Calculations					
	Yb_1	Mo_1	K_1	Sum	V - Sum
O_1	0.913	1.368	0.307	1.98	-0.02
O_2	1.207	1.182		1.79	-0.21
O_3	0.854	1.480		1.91	-0.09
O_4		1.631	0.790	2.03	0.03
Sum	2.97	5.66	1.10		

Bond Lengths			
	Yb_1	Mo_1	K_1
O_1	2.255	1.791	2.826
O_2	2.351, 2.476	1.845	
O_3	2.280	1.762	
O_4		1.726	2.687, 2.784

	Yb-O	Mo-O	K-O
R_0	1.965	1.907	2.132
B	0.37	0.37	0.37

$KLu(MoO_4)_2$

Bond Valence Calculations					
	Lu_1	Mo_1	K_1	Sum	V - Sum
O_1	0.954	1.361	0.324	2.00	0.00
O_2	1.278	1.182		1.82	-0.18
O_3	0.894	1.468		1.91	-0.09
O_4		1.689	0.808	2.09	0.09
Sum	3.13	5.70	1.13		

Bond Lengths			
	Lu_1	Mo_1	K_1
O_1	2.245	1.793	2.805
O_2	2.344, 2.450	1.845	
O_3	2.269	1.765	
O_4		1.713	2.770, 2.683

	Lu-O	Mo-O	K-O
R_0	1.971	1.907	2.132
B	0.37	0.37	0.37
